# Evaluation of microbiological criteria, planktonic communities and trophic state of groundwater resources in Siwa Oasis, Western Desert, Egypt

**DOI:** 10.1038/s41598-025-16167-2

**Published:** 2025-08-24

**Authors:** Mohamad S. Abdelkarim, Mohamed H. H. Ali, Amal A. Othman, Khadiga M. Gaber, Abeer M. A. Mahmoud, Dalia M. Belal

**Affiliations:** 1 Hydrobiology Lab, National Institute of Oceanography and Fisheries (NIOF), Cairo, Egypt; 2 Chemistry Lab, National Institute of Oceanography and Fisheries (NIOF), Cairo, Egypt

**Keywords:** Siwa Oasis, Groundwater, Bacteriology, Phytoplankton, Zooplankton, Ecology, Environmental sciences

## Abstract

Due to their small size and the extreme environmental conditions in hyper-arid regions, wells are highly susceptible to disturbances. Western Desert wells are scarcely studied concerning their ecological status and the environmental factors affecting their aquatic communities. This study aimed to assess groundwater trophic states based on the microbial, phytoplankton and zooplankton composition in three drains and seventeen wells. Principal component analysis, based on physical and chemical parameters, classified the drains and wells into three groups differentiated as; (1) highly mineralized and eutrophic sites with an average conductivity of 12.01 mS/cm. (2) minimally mineralized and eutrophic sites with mean conductivity of 2.14 mS/cm, and (3) an intermediate group with mean conductivity of 7.77 mS/cm. The microbial load exceeded the recommended limits. Fecal coliforms, fecal streptococci and nitrogen-fixing bacteria revealing bacterial contamination in these wells. The studied drains and wells poorly characterized by planktonic species richness. The phytoplankton and zooplankton indices suggested that the ecological status was generally good, with mesotrophic levels. Both phytoplankton and zooplankton communities indicated a minimal disturbance in the drains and wells of Siwa Oasis, while the microbial characterization revealed that these ecosystems are vulnerable and affected by the human activities. This multidisciplinary approach of our study provides new ecological insights into groundwater systems in arid regions like Siwa Oasis. Our work offers baseline ecological data, identifies early warning indicators of water quality degradation, and contributes to a deeper understanding of the interaction between biological communities and groundwater quality.

**Supplementary Information:**

The online version contains supplementary material available at 10.1038/s41598-025-16167-2.

## Introduction

Reliance on groundwater has become an absolute necessity insight of the freshwater scarcity, especially in arid and semi-arid areas, where people depend on groundwater for various agricultural and domestic activities^1,2^. The progressive urbanization, excessive agriculture development and rapid industrialization severely affect the characteristics of groundwater^3^. The dissolved solutes from either geological rocks or anthropogenic origin shared in deterioration of groundwater characteristics^4^. The excessive increase of dissolved solutes from rocks erosion, saline water intrusion, agricultural irrigations, and improper human activities– has significantly worsened groundwater. This phenomenon not only threatens the availability of safe drinking water in arid region, but also has drastic consequences for agricultural, reducing crop yields and diminishing soil fertility^5^.

Combining different biological key elements in aquatic ecosystems bioassessment offers a more effective approach for water quality classification^6^. Living organisms highly response to environmental changes, making them reliable indicators of the aquatic ecosystem health^7^. Different groups of organisms significantly vary in their response patterns to stress impacts and in their effectiveness at detecting various degrees of ecosystem impairments^8^. Bacteria play crucial roles in sustaining the stability of the ecosystems^9^. Distributions of bacterial species primarily depend on nutrient and organic matter loads. They can influence human and animal health, and even may lead to loss of aquatic organisms, but they are valuable indicators of water contamination with human activities and animal waste^10^. The presence of fecal coliforms in any aquatic ecosystem indicates water contamination and suggests the potential presence of other waterborne pathogens^11^. Availability of N_2_–fixing bacteria play an essential role in regulating primary production in aquatic environment and are appreciated indicators of land use effluents to the aquatic systems^12^.

Phytoplankton growth is a primary symptom of eutrophication due to their direct link and sensitivity to nutrient loading^13^. It has traditionally been used to monitor water quality in both lentic, deeper reservoir, and lotic systems^14,15^. It can provide a precisive expression of the environmental health and biological integrity in aquatic ecosystem^16^, making them appreciated indicators for evaluating of water impairment^17^. Phytoplankton functional classification is being progressively used in ecological research to understand the community-environmental relationship^18^, providing a more precisive evaluation of the variations in the aquatic environment^19^. Rotifers are omnipresent organisms, inhabit all types of water, from large reservoir and lakes to small wells and springs, from acidic mining lakes to the open seas, from hyper-oligotrophic lakes to sewage ponds, and from freshwater to hypersaline ponds^20^. Rotifers are very sensitive to environmental stress^21^ and changes in trophic state^22^ and both species composition and biomass are closely tied to changes in total nitrogen (TN), total phosphorus (TP), and chlorophyll-a levels^23,24^. Moreover, rotifer indices (such as taxonomic and functional groups indices) showed a valuable association with phosphorus and nitrogen fluctuations^25,26^.

This study aims to (1) comparing the phytoplankton and zooplankton classification of drains and wells with that of chemical and physical characters, (2) To what extent is groundwater impaired with the infiltration of polluted surface water? (3) evaluation of the drains and wells ecological status based on phytoplankton and zooplankton indices.

## Results and discussion

### Physico-chemical criteria

The concentrations of various physico-chemical characteristics of the selected sites, with drinking standard limits of world health organization^27^ are shown in (Table 1). Water of selected samples are falls within standard pH limits (6.5-9.0), with pH values ranging from 7.28 to 8.75 indicating alkaline nature, except for W07, which has a slightly acidic pH side with pH of 6.85. Water temperature ranged between 16.01 and 26.77 °C. however, certain wells in Siwa Oasis contain naturally warm water, with values reaches 42.69 °C recorded in W16, exceeding the standard limits of 35 °C.

Total dissolved solids (TDS), salinity and electrical conductivity (EC) exhibited similar distribution pattern across the sampled wells due to their direct relationship^28^. TDS values ranged from 160 to 10,240 mg/l, salinity ranged between 0.14 and 9.63‰, and EC ranged from 0.31 to 16.34 mS/cm. According to Todd and Mays’s classification^29^ classify the groundwater according to TDS values into fresh (0.25–1.0 g/l), brackish (1–10 g/l) and saline (10–100 g/l). based on this classification, only 17.6% of the wells qualify as fresh, while the remaining 82.4% fall into the brackish category. Consequently, TDS values in most wells exceed the recommended limits for both drinking (1000 mg/L) and irrigation water (1500 mg/l) purposes. Turbidity values were within acceptable limits, varying from 0.3 to 13.1 NTU. Dissolved oxygen (DO) levels generally higher in cooler wells, averaging around 4.0 mg/l. In contrast, wells with warmer showed lower DO contents particularly in W16, where DO dropped to 1.2 mg/l at a temperature of 42.69 °C.

Biological oxygen demand (BOD) and chemical oxygen demand (COD) values fluctuated within narrow range (0.2-2.0 and 0.6–18.2 mg/l respectively) and remained within the permissible levels for drinking and irrigation purposes. Nitrite (NO_2_^−^-N) values were slightly increased in drainage water compared to groundwater from wells, ranging from 1.77 to 16.92 µg/l. nitrate (NO_3_^−^-N) contents varied widely between 169.3 and 1562.8 µg/l, with elevated levels at W16 and W06 attributed to anthropogenic sources such as agricultural wastes, animal waste and soil leaching^30^. Ammonium (NH_4_^+^-N) values ranged from of 39.2–275.2 µg/L. It is clearly observed that, ammonium is lower than nitrate concentration suggests active nitrification process, especially in presence of DO. The values of PO_4_^3−^-P and TP-P values distributed similarly in the range of (9.0–240.5 and 44.8–1202.7 µg/l, respectively).

### Wells classification based on physical and chemical characteristic

**Fig. 1:**
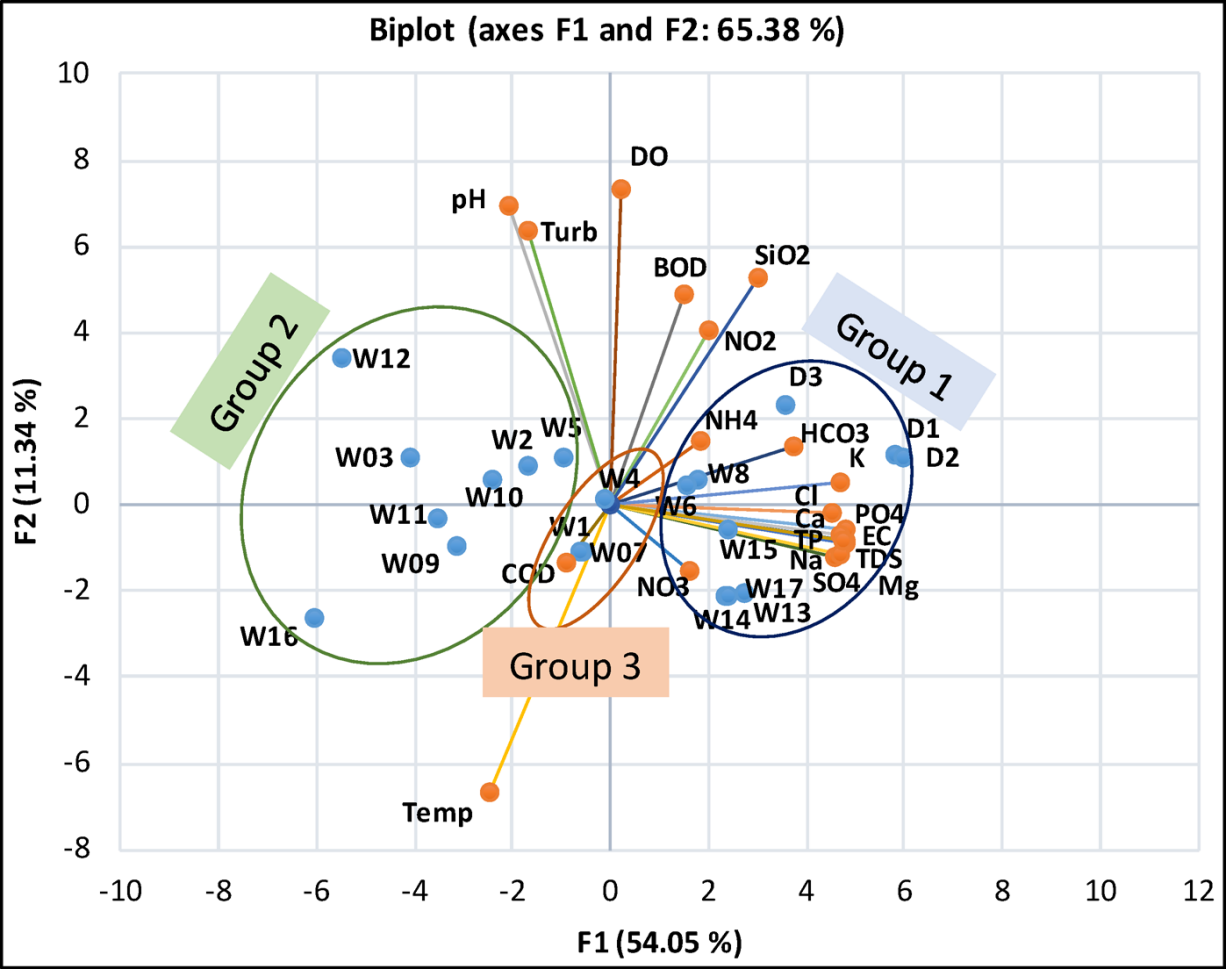
Principal component analysis of different drains and wells based on physical and chemical variables, W: (Wells, 1–17), D (Drains 1–3), DO (Dissolved Oxygen), BOD (Biological Oxygen Demand), COD (Chemical Oxygen Demand), SiO_2_-Si (Reactive silicate), HCO_3_^−^ (Bicarbonate alkalinity), NO_3_^−^-N (Nitrate), NH_4_^+^-N (ammonium), Turb. (Turbidity), NO_2_^−^-N (Nitrite), Temp (Temperature), PO_4_-P (orthophosphate), Cl^−^ (Chloride), TDS (Total Dissolved Solids), Na^+^ (Sodium), K^+^ (Potassium), EC (Electrical conductivity), TP-P (Total phosphorus), Mg^+ 2^ (Magnesium).

PCA biplot of the physical and chemical variables for the samples is shown in (Fig. 1). PCA axes explain a total of 54.04% and 11.34% of the variance in the environmental data, respectively. The highly mineralized, eutrophicated and carbonated wells and drains are positioned at the right side of the factorial plane (Group 1). This group includes the three drains and wells numbered 6, 8, 13, 14, 15 and 17 with a mean conductivity of approximately 12.0 mS/cm and high concentration of sodium, potassium, calcium, magnesium, chloride, sulphate, total inorganic nitrogen (TIN; NO_3_^−^-N **+** NO_2_^−^N **+** NH_4_^+^-N) and total phosphorus (TP-P). Average concentrations for this group are: Na^+^ (1492 mg/l), K^+^ (219 mg/l), Mg^2+^ (700 mg/l), Ca^2+^ (410 mg/l), Cl^−^ (3293 mg/l), HCO_3_^−^ (213 mg/l), TIN-N (771 µg/l), TP-P (1004 µg/l), SO_4_^2−^ (1015 mg/l), and SiO_2_-Si (5.2 mg/l). At the left side of the factorial plane (Group 2), are the less mineralized and eutrophicated wells (mean conductivity was 2.14 mS/cm). This group includes the wells of numbered 2, 3, 5, 9, 10, 11, 12, and 16, with average concentrations of: Na^+^(458 mg/l), K^+^ (72 mg/l), Mg^2+^ (130 mg/l), Ca^2+^ (93 mg/l), Cl^−^ (842 mg/l), HCO_3_^−^ (106 mg/l), TIN-N (354.0 µg/l), TP-P (311 µg/l), SO_4_^2−^ (240 mg/l), and SiO_2_-Si (2.7 mg/l). The intermediate group (Group 3) is located at the middle of the factorial plane and includes wells numbered 1, 4, and 7. This group is characterized by mean conductivity of 7.77 mS/cm, with average concentration of: Na^+^ (1091 mg/l), K^+^ (156 mg/l), Mg^2+^ (261 mg/l), Ca^2+^ (259 mg/l), Cl^−^ (2144 mg/l), HCO_3_^−^ (130 mg/l), TIN-N (403 µg/l), TP-P (563 µg/l), SO_4_^2−^ (596.6 mg/l), and SiO_2_-Si (1.58 mg/l).

**Table 1:** Mean concentrations of physical and chemical characteristics in the main agricultural drains and some Wells in Siwa Oasis, (D_1 − 3_) and wells (W_1 − 17_).

Samples no.	Hydrographic measurements						Oxygen measurements			Major anions (mg/L)			Major cations (mg/L)				Nutrient salts					
	pH	Temp °C	EC mS/cm	Sal (‰)	Turb. (NTU)	TDS g/L	DO mg/L	BOD mg/L	COD mg/L	HCO_3_^−^	SO_4_^2−^	Cl^−^	Ca^2+^	Mg^2+^	Na^+^	K^+^	NO_2_^−^ -*N* µg/L	NO_3_^−^-*N* µg/L	NH_4_^+^- *N* µg/L	PO_4_ ^− 3^ - *P* µg/L	TP-*P* µg/L	SiO_2_-Si mg/L
Agricultural drains																						
D1	7.53	18.36	13.93	8.42	4.4	9.21	2.4	1.8	12.0	210	1103	4325	725	950	1681	301	16.9	208.6	225.3	240.5	1202.7	6.7
D2	7.75	17.90	16.34	9.63	1.2	10.24	4.0	1.8	5.0	300	1031	5018	517	998	2014	339	4.5	484.3	28.8	206.1	1030.5	8.6
D3	7.80	17.81	11.31	6.36	1.2	6.77	6.0	2.0	2.6	205	998	3061	417	631	1344	215	6.1	872.7	55.0	219.5	1097.6	7.5
Groundwater wells																						
W01	7.65	23.60	8.26	4.59	0.3	4.74	2.8	1.6	16.2	140	498	2323	273	224	1218	148	2.8	194.1	147.1	119.7	598.4	1.1
W02	7.72	25.81	6.69	3.60	4.4	3.91	4.4	1.4	9.0	120	439	1789	120	215	1032	116	3.5	251.0	64.7	87.7	438.5	2.0
W03	7.95	26.77	1.71	0.86	1.0	0.93	4.0	2.2	4.6	100	115	317	80	58	171	64	6.9	304.5	45.3	25.1	125.3	2.6
W04	7.81	18.35	8.47	4.74	2.8	5.12	3.2	1.4	4.6	125	755	2282	281	346	1072	184	4.9	236.8	113.3	111.0	555.0	1.0
W05	7.70	17.75	6.89	3.40	5.2	3.73	3.6	1.6	7.2	150	582	1560	200	280	857	110	7.0	284.0	46.6	116.3	581.6	3.2
W06	7.75	16.52	10.36	5.80	4.6	6.33	3.6	1.2	11.8	230	1131	2549	317	509	1298	198	4.7	211.5	47.0	186.2	930.9	4.2
W07	6.81	23.66	6.57	3.60	2.3	3.99	3.2	1.6	18.2	125	536	1827	224	214	982	136	3.8	231.9	275.2	107.3	536.4	2.7
W08	7.79	16.01	8.44	4.73	1.0	5.16	3.6	1.0	8.6	325	699	2127	161	424	1154	158	2.2	1065.4	24.3	179.7	898.6	7.0
W09	7.82	24.52	3.20	1.67	0.5	2.08	2.4	1.2	10.8	115	152	964	74	157	465	86	1.8	222.4	81.1	88.3	441.7	2.5
W10	8.23	18.58	5.15	2.79	1.0	3.15	4.0	0.8	7.6	75	347	1418	160	146	756	118	3.7	169.3	41.5	89.3	446.6	3.0
W11	7.92	24.92	2.69	1.39	2.7	1.58	2.8	1.2	10.6	125	255	609	88	141	318	57	2.4	449.9	70.3	71.0	355.0	2.7
W12	8.75	18.66	0.50	0.24	13.1	0.27	3.2	1.2	5.4	75	25	65	16	27	43	15	5.8	376.2	64.4	9.0	44.8	3.8
W13	7.44	26.14	12.97	7.46	0.3	7.92	2.4	1.4	5.8	155	1275	3325	353	672	1824	233	1.8	181.8	48.9	195.0	975.2	2.9
W14	7.49	23.91	11.75	6.70	0.6	7.18	1.6	1.2	0.6	175	987	3145	361	792	1384	172	5.4	827.4	51.9	197.5	987.4	1.8
W15	7.70	22.29	11.49	6.36	1.1	6.88	2.4	1.2	10.2	160	979	3045	361	668	1388	183	7.7	576.2	65.6	195.7	978.6	6.2
W16	7.61	42.69	0.31	0.14	2.7	0.16	1.2	0.2	10.6	90	9	13	8	15	20	9	2.7	288.0	39.2	10.7	53.6	1.8
W17	7.28	24.26	11.52	6.37	1.4	7.02	2.4	1.2	0.6	160	931	3045	477	658	1341	169	4.7	1562.8	43.8	186.2	930.9	2.2

Kruskal–Wallis tests were used to assess significant differences in physical and chemical variables among the three groups identified by PCA. The results showed significant difference for most variables; Na^+^ (*P* < 0.001), K^+^ (*P* < 0.0001), Mg^2+^ (*P* < 0.005), Ca^2+^ (*P* < 0.01), Cl^−^ (*P* < 0.005), HCO_3_^−^ (*P* < 0.01), SO_4_^2−^ (*P* < 0.005), SiO_2_-Si (*P* < 0.0001) TIN-N (*P* < 0.001) and TP-P (*P* < 0.001).

### Microbial characteristics

Table 2 presents the microbiological results from The studied groundwater, with ANOVA analysis. The microbial load generally exceeded The recommended limit of 1 × 10^2^ cfu/ml for drinking purposes^31^. Total coliform counts ranged from 0 to 2400 mpn/100 ml, with The majority of The groundwater samples surpassing The FAO^32^ standard of less than 2 mpn/100 ml. Fecal coliforms and fecal Streptococci were found in ranges of 0–240 and 0–2900 mpn/100 ml, respectively. Similar results have been observed in Siwa Oasis due to The high infiltration rates of surface soils and The strong vertical permeability, which facilitate The transfer of bacteria into The miocene aquifer, as well as The preferentially survive of bacteria under The sessile status. The fecal coliforms to fecal Streptococci ratio (FC/FS) was below 0.7 in water samples, indicating that The source of fecal contamination primarily non-human, originating from animals^33^. Nitrogen-fixing bacteria (diazotrophs) ranged between 0.50 × 10^2^ and 7.75 × 10^4^ cfu/ml. The notable presence of different bacterial forms in The groundwater samples suggests ongoing pollution. Fecal coliforms, fecal Streptococci and nitrogen-fixing bacteria, were detected in The studied wells. Total coliform showed significant negative correlations (*r* = − 0.55 and − 0.54, respectively, *n* = 20, *P* < 0.05) with EC and salinity levels, respectively. On The other hand, fecal Streptococci show insignificant correlation with salinity. These results indicate that fecal Streptococci are tolerance to salinity.

**Table 2:** Mean bacterial population of water samples in Siwa Oasis with ANOVA test. *ANOVA* means in the same column followed by the same letter are not significantly different (*P* < 0.05). *NA* not available. W: (Wells, 1–17), D (Drains, 1–3). *FAO^28^, **EC^27^.

Sample no	Total coliform	Fecal coliform	Fecal streptococci	Total bacteria count at 37 °C	Total bacteria count at 22 °C	Total diazotrophs
	MPN / 100 ml	MPN / 100 ml	MPN / 100 ml	cfu / ml ×10^2^	cfu / ml ×10^2^	cfu / ml ×10^2^
Drainage water						
D1	240^ef^	93^c^	1200^e^	25.6^gh^	0.6^d^	8.5 ^hi^
D2	12^f^	4 ^hi^	1100^f^	40.0^fg^	3.4^d^	0.5^i^
D3	23 ^f^	12^gh^	1200 ^e^	25.6^gh^	26.6^cd^	28.3^efgh^
Groundwater						
W01	190 ^f^	43^e^	2900^a^	56.8^ef^	83.2 ^cd^	40.5 ^de^
W02	0.0	0.0	1200 ^e^	7.6 ^h^	11.6 ^d^	6.5 ^hi^
W03	600^d^	15^g^	1200 ^e^	39.6^fg^	60.8 ^cd^	11.0^ghi^
W04	94 ^f^	9^gh^	1100 ^f^	6.0 ^h^	6.6 ^d^	6.0 ^hi^
W05	460 ^de^	240^a^	1100 ^f^	26.6^gh^	30.0 ^cd^	32.5 ^efg^
W06	1100 ^c^	75^d^	1200 ^e^	30.0^gh^	32.0 ^cd^	42.5 ^de^
W07	23 ^f^	6^ghi^	1100 ^f^	6.6 ^h^	5.8 ^d^	1.0^i^
W08	43 ^f^	34^f^	240^g^	10.6 ^h^	9.0 ^d^	21.5^efghi^
W09	15 ^f^	0.0	1200 ^e^	12.0 ^h^	19.8 ^cd^	35.5^def^
W10	1200^c^	12^gh^	53^h^	170.0^c^	122.0^bc^	275.0^c^
W11	15 ^f^	7^ghi^	1600^c^	22.4^gh^	19.4 ^cd^	15.5^fghi^
W12	2400^a^	29^f^	1500^d^	77.6 ^de^	284.0^a^	13.5^fghi^
W13	36 ^f^	4 ^hi^	2900 ^a^	226.0^b^	260.0 ^a^	400.0^b^
W14	14 ^f^	9^gh^	1200 ^e^	8.9 ^h^	12.8 ^d^	43.5 ^de^
W15	23 ^f^	0.0	1200 ^e^	25.4^gh^	12.8 ^d^	16.5^fghi^
W16	1800^b^	150^b^	2400^b^	560.0^a^	214.4 ^ab^	775.0^a^
W17	460^de^	7^ghi^	1500 ^d^	90.8^d^	96.0 ^cd^	58.0^d^
**LSD at 0.05**	**211**	**7.28**	**68.75**	**20.92**	**85.73**	**18.93**
**Drinking water Standard**	**(2 MPN/100ml)** ^*^	**NA**	**NA**	**(100 cfu/ml)** ^**^	**(10 cfu/ml)** ^**^	**NA**

### Phytoplankton

#### Taxonomic composition

A total of 99 phytoplankton species from 5 classes were identified; cyanprokaryotes (47 species), Bacillariophyta (44 species), Chlorophyta (4 species), Dinophyta (3 species) and Prymnesiophyta (1 species) (Table S1). The highest species counts of 29 and 26 species were found in Drain 1 and well 6, respectively, while the lowest counts were found in Drain 3 and well 17, with only 7 species. Beauger et al.^34^ reported species richness in the mineral saline wells in the French Massif Central ranged from just one species at some wells to 67 in others. Low species richness was common feature in many wells with a man-made artificial construction around the emergence sites as they noted.

The most common algal genera were, *Nitzschia* (11), *Oscillatoria* (6), *Spirulina* (4), *Navicula* (4), *Aphanocapsa* (4), and *Microcystis* (4). The species *Gomphosphaeria* sp., *Oscillatoria limnetica*, *Denticula tenuis*,* Mastogloia braunii*, *Nitzschia sigma*,* N*. sp. and *Tetramphora decussata* were recorded in at least half of the investigated wells. Most of these species are not typically planktonic but are known as attached forms, previously recorded in various freshwater habitats in Egypt^35^. The identified phytoplankton species can be divided into 19 phytoplankton functional groups: B, C, D, H1, H, J, K, TC, Lo, M, N, MP, P, S1, S2, TD, X1, X3 and Y, with the groups D, MP, P, and TD contributed the most to the total phytoplankton biomass (shown in Table S1). Several of these phytoplankton functional groups were reported in the deep karst lakes, Croatia^36^.

**Fig. 2:**
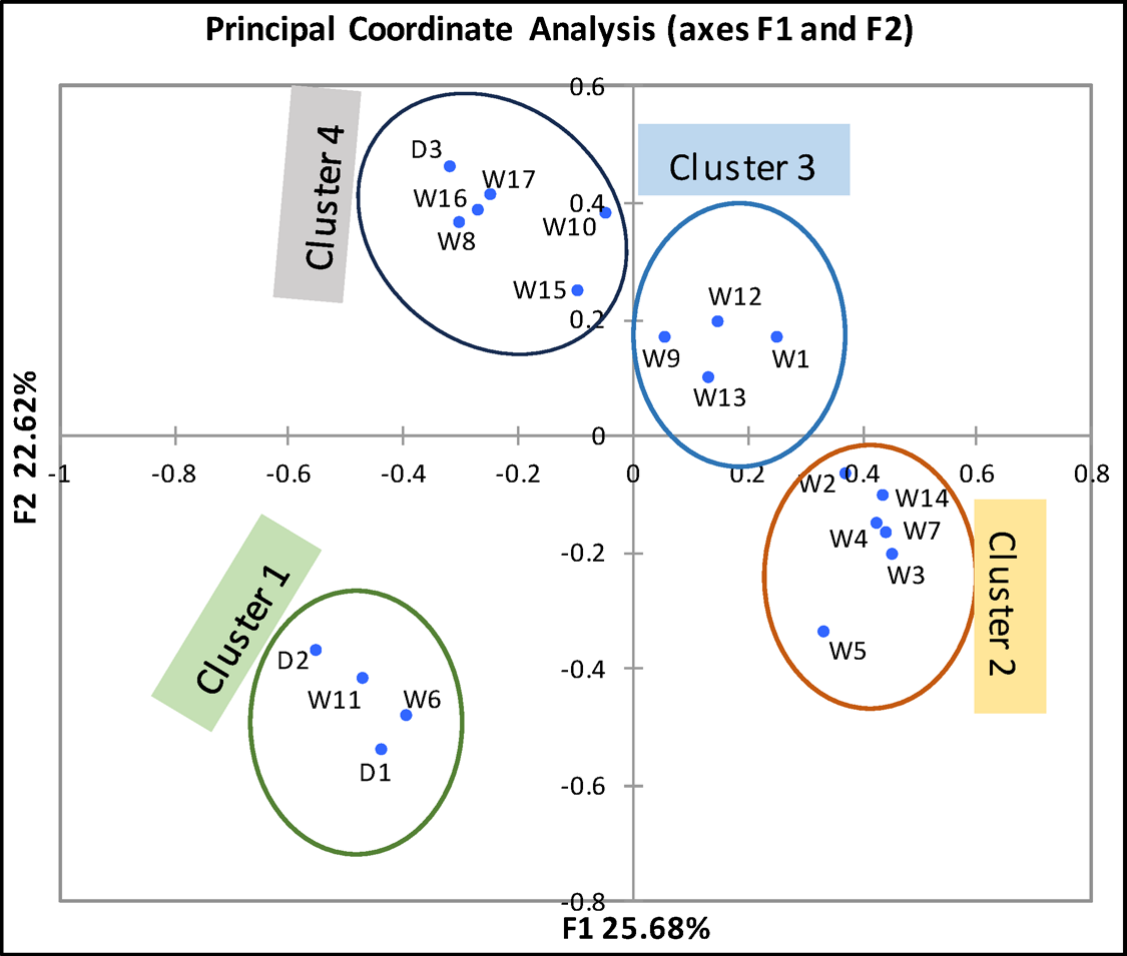
PCoA dendrogram of algal classification based on species composition and abundance at different drains (D_1 − 3_) and wells (W_1 − 17_).

PCoA analysis classified the various drains and wells samples into four distinct clusters based on phytoplankton abundance and species composition (Fig. 2). ANOSIM confirmed significant differences in species composition between these clusters (global *R* = 0.73, *P* < 0.0001). The four algal clusters identified were; cluster 1 (drains 1 and 2 and the wells 6 and 11), cluster 2 (wells 2, 3, 4, 5, 7, and 14), cluster 3 (wells 1, 9, 12, and 13), and cluster 4 (drain 3 and wells 8, 10, 15, 16, and 17).

SIMPER analysis revealed an average dissimilarity of 87% between these groups with *Tetramphora decussata* (25.7%), *Nitzschia sigma* (15.89%), *Denticula tenuis* (13.12%), *Pleurosigma elongatum* (12.36%), *Melosira moniliformis* (10.32%), and *Mastogloia braunii* (5.798%) were the most significant contributed to the differences between groups.

These clusters were obviously different from the groups differentiated by PCA (Fig. 1) based on physical and chemical characteristics, suggesting that additional factors may influence algal composition and abundance. High levels of radioactive nucleotides could play a role and affect the algal assemblages. Abdelkarim and Imam^37^ found that microbial films from Siwa Oasis wells had high concentrations of ^226^Ra, ^232^Th and ^137^Cs, which may affect the composition and abundance of diatoms and other plankton communities. Millan et al.^38^ suggested that the physical and chemical variables might not drive the algal community distribution, while radon nucleotides content of water could be a key driver of the diatoms species composition and the rate of teratological forms.

Indicator value analysis of algal assemblages for different clusters comprised seventeen species with a relative biovolume of ≥ 1% in two sites or more (Table S2). Among these species, only nine species showed significantly different (*p* ≤ 0.01) between the clusters. The indicator species were *Achnanthes armillaris*,* Cerataulina pelagica*,* Denticula tenuis*, *Diploneis oblongella*,* Melosira moniliformis*, *Nitzschia sigma*,* N. palea*,* Pleurosigma elongatum*,* and Tetramphora decussata*. Cluster 1 was characterized by *Melosira moniliformis and Pleurosigma elongatum*, Cluster 2 by *Diploneis oblongella and Tetramphora decussata*, Cluster 3 by *Denticula tenuis*, while Cluster 4 by *Achnanthes armillaris*, *Cerataulina pelagica*, and *Nitzschia sigma.*

#### Phytoplankton speciesenvironmental variables relationships

**Fig. 3:**
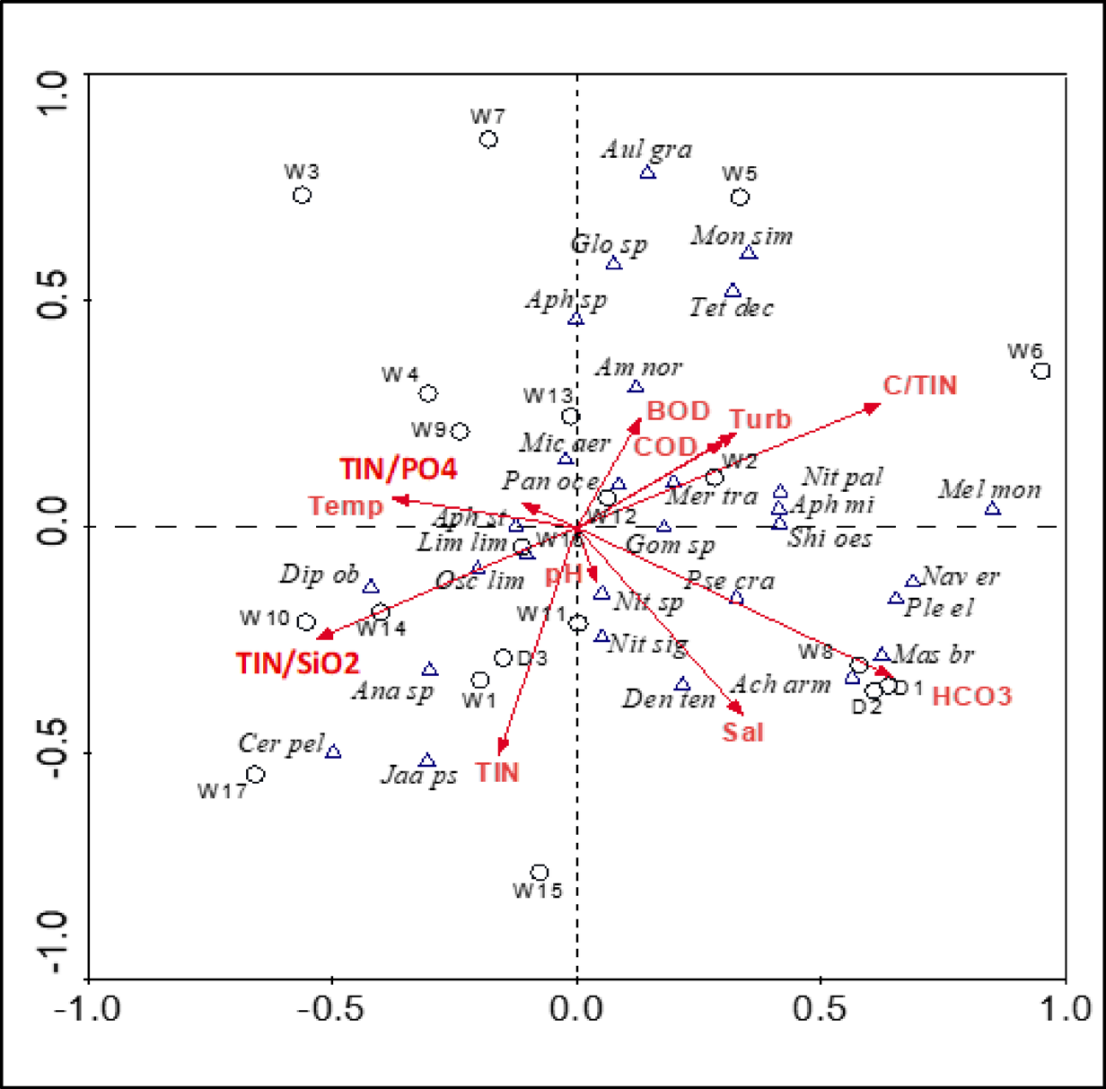
RDA triplot of phytoplankton species (Triangle) and sites (Circle) in relation to some environmental variables (Arrows); W; Well_1 − 17_ and D; Drian_1 − 3_, Species abbreviations are presented in Table S1, while, Sal; Salinity, TIN; Total Inorganic Nitrogen, SiO_2_; Reactive Silicate, HCO_3_^−^; Bicarbonate Alkalinity, Temp; Temperature, PO_4_-P; orthophosphate, Turb; Turbidity, C; Carbon.

The eigenvalues of the first RDA axis (0.214) and the second RDA axis (0.093) were both significant (*P* = 0.01 based on a Monte Carlo permutations test with 499 permutations), and the environmental variables explained 62.8% of the total variance in the species data (Fig. 3). This percentage is very close to 62.1% reported by Lai et al.^39^ for the relation between diatoms and environmental variables in the Mediterranean thermal wells in Sardinia, but significantly higher than the percentage of 18.5% explained by Beauger et al.^33^ for the diatom assemblages in mineral saline springs in the French Massif Central. The species–environment correlation for RDA axis1 (0.921) and RDA axis2 (0.933) indicating a considerable association between selected phytoplankton species and environmental variables. The RDA analysis showed that TIN/SiO_2_, C/TIN, TIN-N, salinity and HCO_3_^−^ are the most important variables in the studied wells, while pH and TIN/PO_4_ are less influential. These results indicated that nitrogen, rather than phosphorus, is the limiting macronutrient affecting the growth of microalgae in the studied well, which contrast with the findings of Lai et al.^39^. The TIN/TP ratio averaged 1.6, with 14 drains and wells were falling below 1.0. W14, exclusively, matched the optimal Redfield^40^ TIN/TP ratio of 7–16. The TIN/TP ratio varied from 0.28 to 9.96. *Anabaena* sp., *Jaaginema pseudogeminatum* and *Cerataulina pelagica* were associated with the highest TIN-N levels and were found in wells 1, 15 and 17 and drain 3. *Navicula erifuga*, *Pleurosigma elongatum*, *Mastogloia braunii*, *Achnanthes armillaris* were strongly linked to HCO_3_ and were highly abundant in W08 and drains 1 and 2. *Aulacoseira granulata*, *Monactinus simplex*, *Tetramphora decussata* and *Gloeocapsa* sp. were closely accompanied with C/TIN and are good characteristics of well 5. Also, *Pseudanabaena limnetica* and *Diploneis oblongella* were highly linked with C/TIN and are good characteristics of W10 and W14. Most of the diatom species associated with TIN-N were selected by the mesotrophic to eutrophic conditions^41^.

### Zooplankton

#### Taxonomic composition

A total of 27 zooplankton species were recorded; Rotifera 24 species, Copepoda one species, and Protozoa two species. In addition to Nauplius larvae, and Copepodite stage of Copepoda and meroplankton stages such as nematoda larvae, chironomus larvae, and nymph of *Enallagma* sp. were also observed. Rotifera was the most dominant group, accounting 80.4% of the total zooplankton density (Table S3). High levels of nitrogen and phosphorus shift zooplankton composition from larger species like Copepoda and Cladocera, to smaller rotifers^42^. Rotifers are cosmopolitan species, influenced more by ecological factors than geographical distribution^43^. Species such *Brachionus calyciflorus*, *Keratella cochlearis* and *Trichocerca* spp. tend to become more abundant as the nutrient levels increase^44^.

**Fig. 4:**
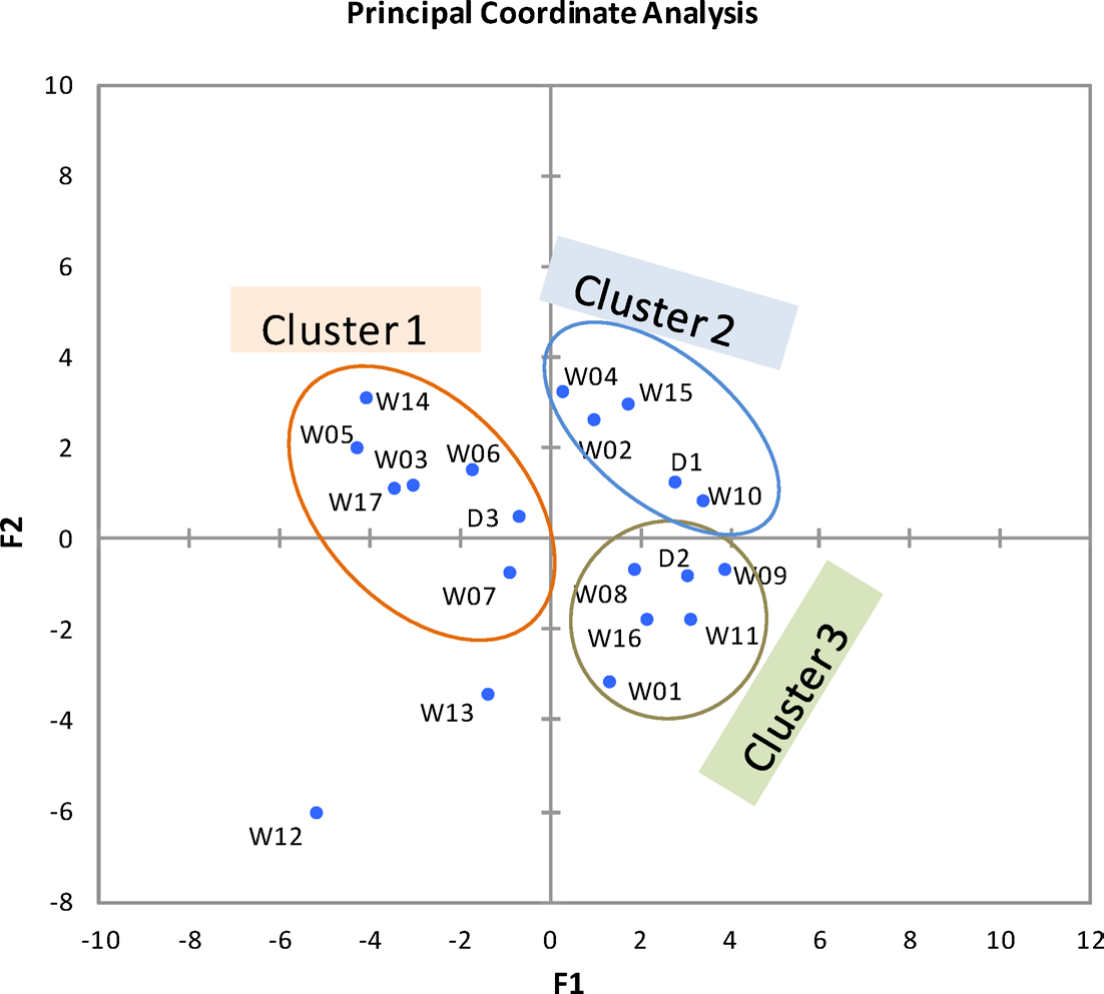
PCoA dendrogram of zooplankton classification based on species composition and abundance at different drains (D1-D3) and wells (W1- W17).

PCoA analysis differentiated the various drains and wells samples based on zooplankton abundance and composition into three distinct clusters (Fig. 4). ANOSIM confirmed the significant differences in species composition between different clusters (global *R* = 0.81, *P* < 0.005). The three identified clusters were: cluster 1 (drain 3 and the wells 3, 5, 6, 7, 14 and 17), cluster 2 (drains 1 and wells 2, 4, 10, and 15), cluster 3 (drain 2 and wells 1, 8, 9, 11, and 16), while wells 12 and 13 highly recognized from these clusters. SIMPER analysis revealed that the groups had an average dissimilarity of 61.52% with the species *Philodina roseola* (17.29%), *Colurella adriatica* (15.34%), *Lecane closterocerca* (11.83%), *Trichocerca elongata* (11.82%), nauplius larvae (7.23%), *K. cochlearis* (7.2%) and Nematoda larvae (6.55%) contributing most significant to the differences between groups.

Indicator value analysis of zooplankton assemblages for different clusters comprised twenty species with a relative biovolume of ≥ 1% in two sites or more (Table S4). Between these species, only eleven species were significantly different (*P* ≤ 0.05) between the four clusters. *Colurella adriatica*,* Conochilus unicornis*,* Hexarthra* sp., *Keratella cochlearis*,* Philodina roseola*,* Lecane bulla*,* L. closterocerca*,* Trichocerca elongata*, Nauplius larvae, Nematoda larvae and Nymph of *Enallagma* sp. were the indicator species between different clusters (Table S4). Cluster 1 was characterized by *C. adriatica*,* K. cochlearis*, Nauplius larvae *and H.* sp., Cluster 2 was categorized by *C. adriatica*,* P. roseola*, Nymph of *Enallagma* sp. *and* Nematoda larvae, Cluster 3 was distinguished by *P. roseola*,* L. bulla*,* L. closterocerca* and Nymph of *Enallagma* sp., while Cluster 4 was characterized by *C. unicornis*, and *T. elongata.*

#### Zooplankton speciesenvironmental variables relationships

**Fig. 5:**
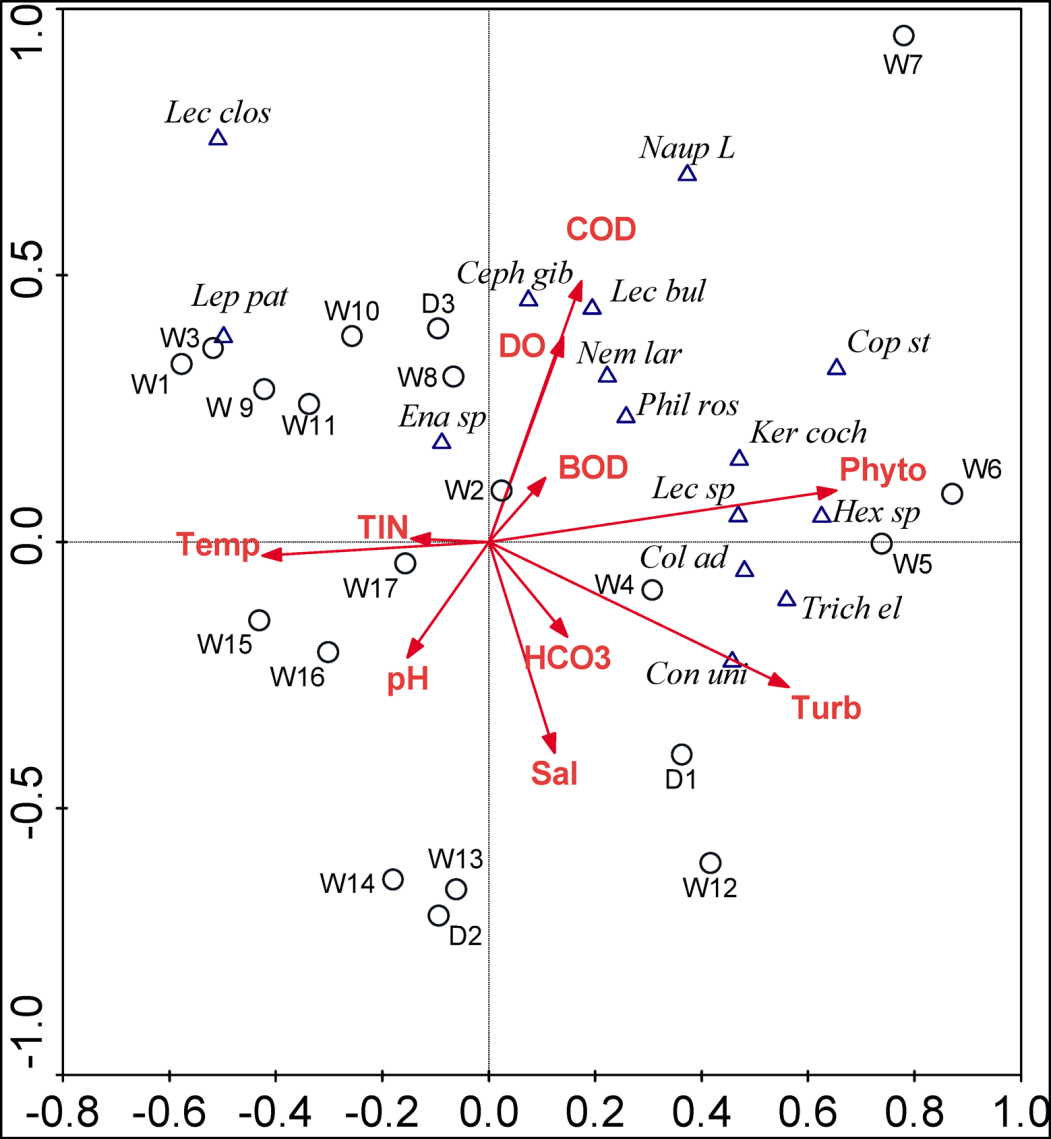
RDA triplot of zooplankton species (Triangle), sites (Circle) in relation to some environmental variables (Arrows); W; Well_1 − 17_ and D; Drian_1 − 3_, Species abbreviation are presented in Table S3, while Sal; Salinity, Temp; Temperature, Tur; Turbidity, HCO_3_; Bicarbonate Alkalinity, Phyto; Phytoplankton Biovolume, TIN; Total Inorganic Nitrogen.

The eigenvalues of the first RDA axis (0.19) and the second RDA axis (0.16) were both significant (*P* < 0.05 based on Monte Carlo permutations test with 499 permutations), with the selected environmental variables explaining 59.9% of the total variance in the species data (Fig. 5). This correlation is very close to the total explained variance (62.8%) observed between phytoplankton species data and the environmental variables in this study, as well as 62.1% reported by Lai et al.^39^ for diatoms and environmental factors in a thermal well in Sardinia. The species–environment association for RDA axis1 (0.941) and RDA axis2 (0.92) indicate a considerable association between selected zooplankton species and environmental variables. The RDA analysis showed that the physical conditions, salinity, turbidity and temperature, are the key drivers of zooplankton distribution in the studied wells compared to the chemical conditions, pH, BOD, and TIN. These results contrast with other studies, which suggested that TIN and TP are key factors influencing rotifer species^45^. Most zooplankton species showed a strong inverse association with salinity, revealing that the zooplankton inoculum in the high mineral and thermal wells is of freshwater origin. Many of these species were recorded in the River Nile^46^ and Lake Nasser^47^. However, *Colurella adriatica*,* Conochilus unicornis* and *Trichocerca elongata* can tolerate wide range of salinity, similar results were reported by Hegab et al.^48^ in Lake Manzala.

#### Wells tropic state assessment based on phytoplankton assemblage index (Q) and rotifer indices (TSI_Rot_)

The application of Q and TSI_Rot_ indices for assessing the quality of drains and different wells are presented in (Table 3). Both indices revealed similar ecological conditions and were strongly correlated (*r* = 0.697, *P* < 0.05). TSI_Rot_ classified the ecological status of drains and wells as mesotrophic, while Q index showed more diversified levels. According to the Q index, nine drains and wells were rated as excellent, seven wells as good, and 4 wells and drains as medium. These results are significantly similar to the deductions of Kamberović1 et al.^49^ concerning the thermal springs in Europe. Both TSI_Rot_ and the Q indices showed significant correlation with TIN (0.421 and − 0.435, respectively) and NO_3_ (0.457 and − 0.409, respectively), but neither index was associated with temperature, TP, PO_4_, or various minerals (Table S5). The morpho-functional approach of phytoplasnkton assesses the ecological status of Croatian natural karstic lakes, classifying them as either good or high and confirming their oligotrophic and mesotrophic conditions^50^. Environmental drivers of aquatic ecosystems can be studied through trait-based approaches of phytoplankton^18^. The results of Kuczynska-Kippen et al.^51^ confirmed the great suitability of rotifer indices for environmental studies. Moreover, the variation in rotifer dynamics may be affected by the trophic status of the investigated small water bodies emphasizing their suitability for environmental assessment^43^.

**Table 3:** Wells and drains ecological status assessment based on phytoplankton functional approach (Q index) and rotifer species (TSI_Rot_).

Site	Phytoplankton assemblage index (Q)	Rotifer index (TSI_Rot_)	Site	Phytoplankton assemblage index (Q)	Rotifer index (TSI_Rot_)
D1	Excellent	Mesotrophic	W8	Medium	Mesotrophic
D2	Excellent	Mesotrophic	W9	Good	Mesotrophic
D3	Medium	Mesotrophic	W10	Good	Mesotrophic
W1	Good	Mesotrophic	W11	Excellent	Mesotrophic
W2	Excellent	Mesotrophic	W12	Good	Mesotrophic
W3	Excellent	Mesotrophic	W13	Medium	Mesotrophic
W4	Excellent	Mesotrophic	W14	Good	Mesotrophic
W5	Excellent	Mesotrophic	W15	Good	Mesotrophic
W6	Excellent	Mesotrophic	W16	Medium	Mesotrophic
W7	Excellent	Mesotrophic	W17	Good	Mesotrophic

## Materials and methods

### Site description

**Fig. 6:**
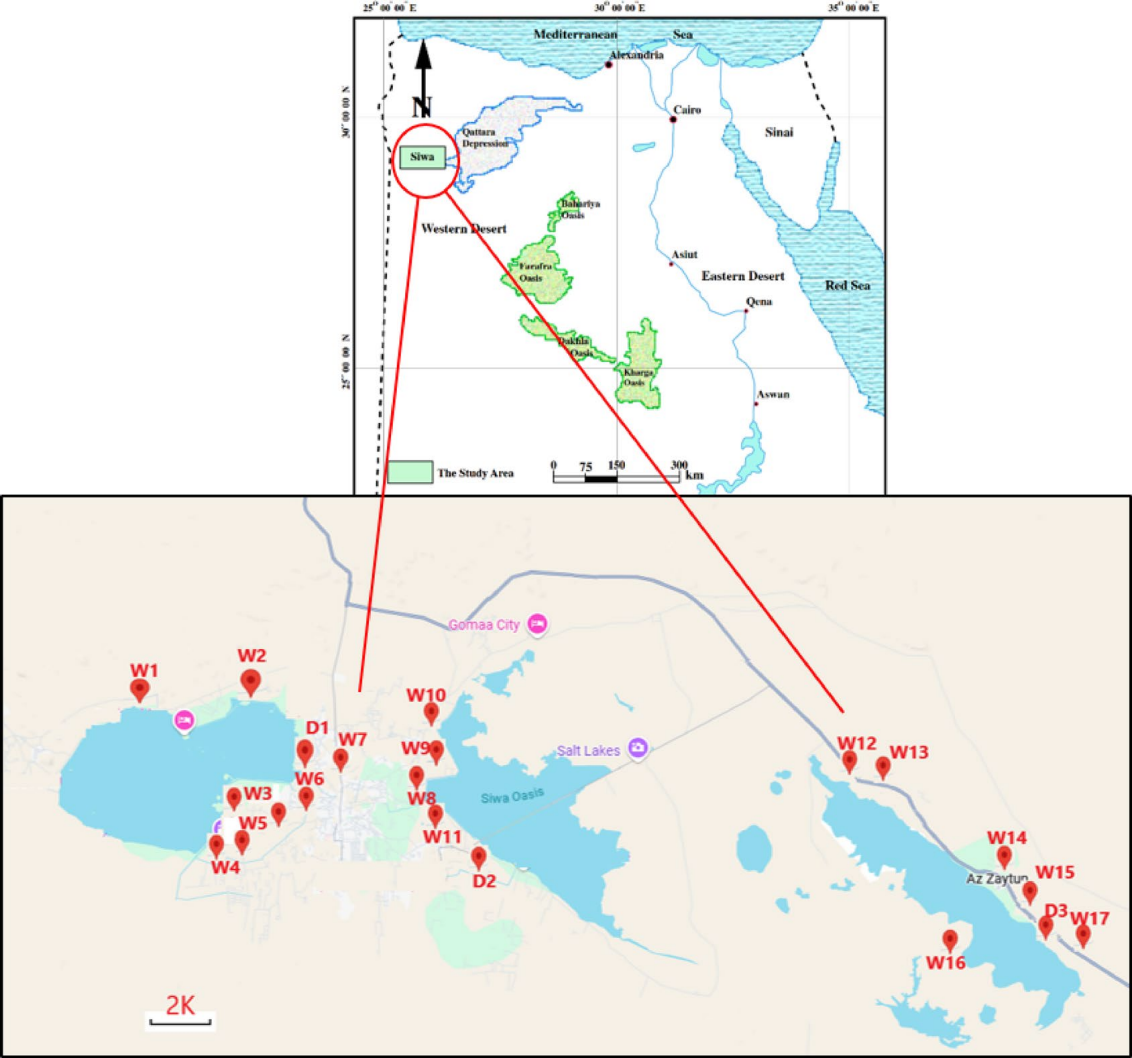
Map showing the sampling sites from some drains and wells in Siwa Oasis, W; Wells 1–17 and D; Drains 1–3.

Siwa Oasis is one of Egypt’s most renowned and ancient urban oases. It situated approximately − 23 m below sea levels, it lies between Great Sand Sea and Qattarah Depression in the Western Desert, about 50 km from eastern Libyan-Egypt borders and 750 km from Cairo^52^. The oasis has irregular elongated shape covering a total area around 1100 km^2^. It stretches roughly 80 km from east to west, with width ranged between 9 and 28 km. Only about 5% of this area is used for cultivation, while the majority is occupied by saline lakes, sabkhas and salt marches (Fig. 6 and Table S6). The most studied wells are artificial constructions. The water of the artificial wells is highly transparent, used for irrigation through concrete channel and occasionally used for recreational purposes^52^. Due to the high light penetration, a thick layer of dense microbial films forms on the bottom. When these layers become thicker and the lower microflora die, they detach, forming dense, floating microbial mats.

### Sampling program and water sampling

Eighty subsurface water samples were collected from three main agricultural drains and seventeen wells across Siwa Oasis over four successive seasons, spanning winter to autumn, 2023 (Fig. 6). A 2.5 L Ruttner water sampler was used for water samples collection. Water samples for chemical, physical, and plankton analysis were collected ensuring they were taken away from any floating microbial mats and two meters from the margin, subsurface water samples were collected for phytoplankton analysis (500 ml), while 30 L were filtered through a plankton net of 55 μm mesh size for zooplankton analysis. Plankton samples were immediately stored in clean labelled polyethylene bottles and preserved using 4% formaldehyde solution. For bacteriological analysis, unfiltered water samples were manually collected using sterile brown bottles (200 mL), stored at 4 °C. Bacteriological analysis was completed within 48 h.

### Chemical analysis

The physical and chemical parameters of water samples were analyzed following the standards methods of American Public Health Association^53^. In situ, measurements of water temperatures, salinity (‰), turbidity (NTU) electrical conductivity (mS/cm) and pH were taken using a calibrated Multi-Parameters Hanna model (HI9829). Total dissolved solids (TDS) were measured by oven-drying method at 180 °C. Dissolved oxygen (DO) was estimated by modified Winkler method, biological oxygen demand (BOD) was assessed through a 5-days incubation method. Chemical oxygen demand (COD) was determined by potassium permanganate method. Carbonate (CO_3_^2−^), bicarbonate (HCO_3_^−^), calcium (Ca^2+^), magnesium (Mg^2+^), chloride (Cl^−^) were determined using titrimetric method. Sulfate (SO_4_^2−^) was determined using turbidimetric method and measured spectrophotometrically at 420 nm. Sodium (Na^+^) and Potassium (K^+^) concentrations were measured using flame photometer (Jenway Felsted Gi Dunmow Essex). Nutrient salts, including nitrite, nitrate, ammonia, orthophosphate, total phosphorus and silicate were measured colormetrically using Jenway 6800UV/VIS double beam spectrophotometer.

### Microbiological analysis

Total coliforms, fecal coliforms and fecal streptococci were enumerated using the most probable number (MPN) procedure^53^, using lauryl tryptose broth (37 °C ± 0.5 °C at 48 ± 2 h) for total coliforms, EC broth media for fecal coliforms (44.5 °C ± 0.5 °C at 24 ± 2 h) and azide dextrose broth (35 °C ± 0.5 °C at 48 2 h) for fecal streptococci. Total bacteria were enumerated on nutrient agar medium at incubation temperatures of 22 °C and 37 °C using pour plate method. Total diazotrophs were enumerated on N-deficient combined carbon source agar medium^54^. The bacteriology data were subjected to the analysis of variance (ANOVA) and Fisher’s least significance difference (LSD) at a significant level of *P* < 0.05, correlation coefficients and linear regressions among the different parameters were determined using STATISTICA v10 (Statsoft, OK, USA).

### Plankton analysis

For phytoplankton analysis, fifty ml of well-shaken preserved water sample has been poured into the combined chamber of Utermöhl^55^ and let for 3 days for complete sedimentation, the entire sample was identified and counted using inverted microscope at 400× magnification and 1000× when necessary. For well identification, the guides of Dillard^56^, Krammer and Lange-Bertalot^57,58^, Starmach^59^, John and Sheath^60^ were used. Algal biovolume was estimated by approximating the cells to the nearest geometric shape according to Hillebrand et al.^61^ and Vadrucci et al.^62^. Zooplankton were examined under a binocular microscope at magnification of 100× to 400×. Three successive subsamples of 1 ml were drawn after careful mixing using a wide-pipette. The mean count was taken and the results were expressed as the number of organisms per cubic meter and the guides of Foissner and Berge^63^, McCallum^64^ and Negera^65^ were used.

### Wells trophic state evaluation based on biological communities

Phytoplankton species were categorized into functional groups (FGs) based on the classifications proposed by Reynolds et al.^66^ and Padisak et al.^67^. The abundance of each functional group is the summation of the abundances of all species classified within the group. The ecological status of various wells and drains was assessed using the Phytoplankton Assemblage Index (Q) Eq. (1) based on Reynolds’ functional classification^68^. To verify this assessment, Q was correlated with different environmental variables presented in (Table 1). The Rotifer State Index^43^ was also applied to evaluate the trophic states of different wells, the used formula are represented in (Table 4).

1 $$\:Q={\sum\:}_{i=1}^{n}piF$$

where pi = ni/N; ni is the biomass of the i-th functional group; N is the total biomass, (F) is a factor number established for the i-th functional group. Reference values of the (Q) index ranges between 0 and 5, of which 0–1: bad; 1–2: tolerable; 2–3:medium; 3–4: good and 4–5: excellent.

**Table 4:** Formulas of rotifer trophic state index (TSI_ROT_).

Parameter	Formulas
Rotifer numbers (N, ind. L^− 1^)	TSI_N_ = 5.38 Ln(N) + 19.28
Total biomass (B, mg w.wt. L^− 1^)	TSI_B_ = 5.63 Ln(B) + 64.47
Percentage of bacterivores in total numbers (BAC, %)	TSI_Bac_ = 0.23 BAC + 44.30
Ratio of biomass to numbers (B: N, mg w.wt. ind. ^–1^)	TSI_B/N_ = 3.85 (B: N)^−0.318^
Percentage of species indicative of high trophy in the indicative group’s numbers (IHT, %)	TSI_IHT_ = 0.203 IHT + 40.0
Rotifer Trophic State Index (TSI_ROT_) is the mean of the calculated indices	

### Statistical analysis

A detrended multivariate analysis was conducted to examine the relationship between the most frequent phytoplankton species with their abbreviations, highlighted in bold in Table (S1), to environmental variables. A detrended correspondence analysis (DCA) was performed on species composition and functional classification to determine whether linear ordination (Redundancy analysis, RDA), or unimodal ordination (Canonical Corresponding Analysis, CCA), was more appropriate^69^. Redundancy analysis (RDA) was used to relate phytoplankton species composition to environmental variable as the gradient lengths was very short (< 3.0s.d) indicating a linear distribution. The significance of the ordination axes was tested using a Monte Carlo test with 499 permutations. For the ordination analysis, data were transformed to log10. To improve the significance of the multivariate analysis, a multi-collinearity test was performed for twenty fife available environmental data set and their rations. Eleven environmental variables were retained and applied for RDA: temperature, turbidity, pH, BOD, COD, salinity, HCO_3_^−^, total inorganic nitrogen (TIN), TIN/PO_4_ orthophosphate), TIN/SiO_2_, C/TIN.

The most frequent zooplankton species distribution with their abbreviations, highlighted in bold in (Table S3), were explained by RDA as represented above. Ten environmental were applied for RDA analysis after the multi-collinearity test; temperature, turbidity, pH, dissolved oxygen (DO), BOD, COD, salinity, HCO_3_^−^, Total inorganic Nitrogen (TIN), and total phytoplankton biovolume (Phyto).

Principal Coordinates Analysis (PCoA) was performed using the proximity matrix derived from the similarity/dissimilarity matrices of the plankton datasets. Differences between the groups of wells identified by the PCoA were tested using a one-way analysis of similarities (ANOSIM). A similarity percentage analysis (SIMPER) was applied to approximation the intergroup dissimilarity and the percentage involvement of the species to the groups difference. Indicator Species Analysis^70^ was applied to identify characteristic species of each cluster formed by PCoA. The significance of each species’ indicator value was estimated using Monte Carlo permutation tests. To avoid conflicts from the rare species, only species with a relative biovolume of ≥ 1% in two sites or more were included in the analysis regardless of the species frequency between wells. DCA and RDA were conducting using Canoco for windows v4.5, while other analyses were performed using XLSTAT 2016 1.1, Past 4.06 and PC-ORD 5 software.

## Conclusion

This study represents the first integrated evaluation of the water quality in some drains and wells of Siwa Oasis. It provides detailed insights about the surface water infiltration into the groundwater and the evaluation of trophic status assessment based on planktonic criteria. The microbiological analysis revealed ongoing contamination of groundwater with fecal coliforms, fecal streptococci and nitrogen-fixing bacteria through the infiltration of animal wastes and fertilizer-laden irrigation water. The classifications of drains and wells based on planktonic biota was greatly differ from that of the chemical and physical characters revealing that other factors likewise radionucleotides activities and water retention may act as key drivers of these assemblages. Both phytoplankton index (Q) and the rotifer index (TSI_Rot_) were strongly linked, and both classified the trophic states of the wells and drains (mostly) at excellent and (always) mesotrophic level, respectively. Even though, the Q and TSI_Ro_t indices showed a good water quality states, while the bacterial loads demonstrated a considerable effect of infiltration of polluted water. The results of this study indicated that the groundwater of Siwa Oasis is vulnerable to surface water contamination and the continuous monitoring is highly recommended.

## Supplementary Information

Below is the link to the electronic supplementary material.

Supplementary Material 1

## Data Availability

All data generated and analysed during this study are included in the manuscript and are available from the corresponding author upon request.
