# Supplementary material for: Evaluation of microbiological criteria, planktonic communities and trophic state of groundwater resources in Siwa Oasis, Western Desert, Egypt
Source: Sci Rep. 2025 Aug 24;15:31090. doi: 10.1038/s41598-025-16167-2 (PMC12375732; doi:10.1038/s41598-025-16167-2)
Supplement: Supplementary file 1 — Supplementary Material 1 [file 41598_2025_16167_MOESM1_ESM.docx]

**Supplementary data**

**Evaluation of Microbiological Criteria, Planktonic Communities and Trophic State of Groundwater Resources in Siwa Oasis, Western Desert, Egypt**

Mohamad S. Abdelkarim^1^, Mohamed H.H. Ali^2*^, Amal A. Othman^1^, Khadiga M. Gaber^1^, Abeer M. A. Mahmoud^1^, Dalia M. Belal^1^

*Corresponding author Email: mhha_ali@yahoo.com

1. Hydrobiology Lab, National Institute of Oceanography and Fisheries, Cairo, Egypt

2. Chemistry Lab, National Institute of Oceanography and Fisheries, Cairo, Egypt

ORCIDs

Mohamad S. Abdelkarim: [0000-0002-8570-3578](https://orcid.org/0000-0002-8570-3578)

Mohamed H.H. Ali: 0000-0003-0141-7791

Aml Othman: 0000-0001-8864-4553

Dalia M. Belal: 0000-0001-9998-1926

Khadeja Mahmoud: 0000-0001-5966-028X

Abeer M.A. Mahmoud: 0000-0003-0628-0915

| Abbreviation of species used in the RDA analysis | Codon of functional groups | Phytoplankton Species | **D1** | **D2** | **D3** | **W1** | **W2** | **W3** | **W4** | **W5** | **W6** | **W7** | **W8** | **W9** | **W10** | **W11** | **W12** | **W13** | **W14** | **W15** | **W16** | **W17** |
| --- | --- | --- | --- | --- | --- | --- | --- | --- | --- | --- | --- | --- | --- | --- | --- | --- | --- | --- | --- | --- | --- | --- |
|  |  | **Cyanophyceae** |  |  |  |  |  |  |  |  |  |  |  |  |  |  |  |  |  |  |  |  |
| **Ana sp** | *H1* | ***Anabaena* sp.** |  |  |  | **0.37** |  |  |  |  |  |  |  | **0.18** | **0.11** |  |  |  |  | **0.15** |  |  |
|  | *H* | *Anathece* *clathrata* (West & G.S.West) Komárek, Kaštovský & Jezberová |  |  |  |  |  |  |  |  | **4.49** |  |  |  |  | **0.41** |  |  |  |  |  |  |
| **Aph sp** | *K* | ***Aphanocapsa* sp.** |  |  |  |  |  |  |  | **0.22** |  | **0.19** |  |  |  |  |  | **1.17** |  |  | **1.16** |  |
|  | *K* | *Aphanothece elabens* (Meneghini) Elenkin |  |  |  |  |  |  |  |  |  |  |  |  |  | **1.59** |  |  |  |  |  |  |
| **Aph mi** | *K* | ***A. microscopica* Nägeli** | **0.82** |  |  |  | **0.20** |  |  |  | **1.09** |  |  |  |  | **0.81** | **3.23** | **1.30** |  |  | **2.82** |  |
| **Aph st** | *K* | ***A. stagnina* (Sprengel) A.Braun** |  |  |  |  |  |  |  | **0.02** |  |  |  |  |  |  |  | **1.63** | **4.24** | **0.03** |  |  |
|  | *K* | *A.* *naegelii* Wartmann | **1.21** |  |  |  |  |  |  |  |  |  |  |  |  | **0.94** |  |  |  |  |  |  |
|  | *S2* | *Borzia* *trilocularis* Cohn ex Gomont |  |  |  |  |  |  |  |  |  |  |  |  |  | **0.09** |  |  |  |  |  |  |
|  | *S2* | *Chamaesiphon* *carpaticus* Starmach |  |  |  | **0.01** |  |  |  |  |  |  |  |  |  |  |  |  |  |  |  |  |
|  | *Lo* | *Chroococcus minor* (Kützing) Nägeli |  |  |  |  |  |  |  |  |  |  |  |  |  |  | **0.02** |  |  | **0.16** |  |  |
|  | *Lo* | *C. minutus* (Kützing) Nägeli |  |  |  |  | **2.83** |  |  |  |  |  |  |  |  |  |  |  |  | **0.12** |  |  |
| **Glo sp** | *MP* | ***Gloeocapsa* sp.** |  |  |  |  |  |  |  | **0.06** |  | **0.03** |  |  |  |  | **0.02** | **0.02** |  |  |  |  |
|  | *MP* | *G. sanguinea* (C.Agardh) Kützing |  |  |  |  | **0.42** |  |  |  |  |  |  |  |  |  |  |  |  |  |  |  |
|  | *MP* | *Gloeothece* *rupestris* (Lyngbye) Bornet |  |  |  |  |  |  | **0.10** |  |  |  |  |  |  |  |  |  |  |  |  |  |
| **Gom sp** | *Lo* | ***Gomphosphaeria* sp.** |  | **1.23** | **1.84** |  |  |  |  | **0.92** | **0.55** | **1.84** |  |  |  | **16.13** | **1.34** | **1.96** | **7.43** | **19.32** |  |  |
| **Jaa ps** | *MP* | ***Jaaginema pseudogeminatum* (G.Schmid) Anagnostidis & Komárek** | **0.08** |  |  |  |  |  |  |  |  |  |  | **3.21** |  |  |  |  | **0.36** |  | **1.01** |  |
|  | *MP* | *Johannesbaptistia pellucida* (Dickie) W.R.Taylor & Drouet |  |  |  |  |  |  |  |  |  |  |  |  |  |  |  |  | **0.02** |  |  |  |
| **Lim lim** | *Lo* | ***Limnococcus limneticus* (Lemmermann) Komárková, Jezberová, O.Komárek & Zapomelová** | **0.01** |  |  |  |  | **0.01** |  |  |  |  | **0.12** | **0.01** | **0.19** |  |  |  |  |  |  |  |
|  | *MP* | *Limnoraphis birgei* (G.M.Smith) J.Komárek, E.Zapomelová | **0.49** |  |  |  |  |  |  |  |  |  |  |  |  |  |  |  |  |  |  |  |
|  | *S1* | *Limnothrix pseudominima* (Skuja) I.Umezaki & M.Watanabe |  |  |  |  |  |  | **1.64** |  |  |  |  |  |  |  |  |  |  |  |  |  |
|  | *MP* | *Lyngbya martensiana* Meneghini ex Gomont |  |  |  |  |  |  |  |  | **0.52** |  |  |  |  |  |  |  |  |  |  |  |
|  | *MP* | *L.* sp. |  |  |  |  | **0.66** |  |  |  |  |  |  |  |  |  |  |  |  |  |  |  |
|  | *Lo* | *Merismopedia glauca* (Ehrenberg) Kützing | **0.04** |  |  |  |  |  |  |  |  |  |  |  |  |  |  |  |  |  |  |  |
|  | *Lo* | *M. tenuissima* Lemmermann |  |  |  |  |  |  |  |  | **0.02** |  |  |  |  |  | **0.02** |  |  |  |  |  |
| **Mer tra** | *Lo* | ***M. tranquilla* (Ehrenberg) Trevisan** |  |  | **0.01** |  |  |  |  |  | **0.03** |  |  |  |  |  |  | **0.06** | **0.01** |  |  |  |
|  | *MP* | *Microcoleus* *attenuatus* (Fritsch) Strunecky, Komárek & J.R.Johansen | **0.26** |  |  |  |  |  |  |  |  |  |  |  |  |  |  |  |  |  |  |  |
| **Mic aer** | *M* | ***Microcystis aeruginosa (Kützing) Kützing*** |  |  |  |  | **0.13** |  |  |  |  | **0.25** |  |  |  |  | **0.89** |  |  | **2.09** |  |  |
|  | *M* | *M. endophytica* (G.M.Smith) Elenkin |  |  |  |  |  |  |  | **0.35** |  |  |  |  |  |  |  |  |  |  |  |  |
|  | *M* | *M. marginata* (Meneghini) Kützing | **1.59** |  |  |  |  |  |  |  |  |  |  |  |  |  |  |  |  |  |  |  |
|  | *M* | *M. viridis* (A.Braun) Lemmermann |  |  |  |  |  |  |  | **0.31** |  |  |  |  |  |  | **0.14** | **0.13** |  |  |  |  |
|  | *H1* | *Nostoc* sp. |  |  |  |  | **0.04** |  |  |  |  |  |  |  |  |  | **0.17** |  |  |  |  |  |
|  | *MP* | *Oscillatoria formosa* Bory ex Gomont |  |  |  |  | **1.62** |  |  |  |  |  |  |  |  |  |  | **0.52** |  |  |  |  |
|  | *MP* | *O. janus* Skuja | **0.49** |  |  |  |  |  |  |  |  |  |  |  |  |  |  |  |  |  |  |  |
| **Osc lim** | *MP* | ***O. limnetica* Lemmermann** | **0.18** |  | **0.07** | **0.21** | **1.38** | **0.23** | **0.26** | **0.11** |  |  | **0.03** | **0.29** | **0.72** | **1.47** |  |  | **0.01** | **0.12** | **2.33** |  |
|  | *MP* | *O. princeps* Vaucher ex Gomont |  |  |  |  |  |  |  |  |  |  |  |  |  |  |  |  |  | **18.84** |  |  |
|  | *MP* | *O. rupicola* (Hansgirg) Hansgirg ex Forti |  |  |  |  |  |  |  |  | **0.33** |  |  |  |  |  |  |  |  |  |  |  |
|  | *MP* | *O. trichoides* Szafer |  |  |  |  |  |  |  |  | **0.46** |  |  |  |  |  |  |  |  |  |  |  |
|  | *MP* | *Phormidium granulatum* (N.L.Gardner) Anagnostidis |  |  |  | **2.62** |  |  |  |  |  |  |  |  |  |  |  |  |  | **1.04** |  |  |
|  | *MP* | *P.* sp. |  |  |  |  |  |  |  |  |  |  |  |  | **1.03** |  |  |  |  | **0.51** |  |  |
|  | *S1* | *Planktolyngbya* *limnetica* (Lemmermann) Komárková-Legnerová & Cronberg |  |  |  |  |  |  |  |  |  |  |  |  |  |  |  |  | **0.12** |  |  |  |
| **Pse cra** | *S1* | ***Pseudanabaena crassa* Uherkovich, nom. Illeg** | **0.07** |  |  |  | **1.67** |  |  |  |  |  | **0.07** |  |  | **0.82** | **0.06** |  |  |  | **0.12** |  |
|  | *S1* | *P. minima (G.S.An) Anagnostidis* |  |  |  |  |  |  |  |  |  |  |  |  |  | **5.25** |  |  |  |  | **0.34** |  |
|  | *S2* | *Spirulina flavovirens* Wisłouch |  |  |  |  |  | **0.93** |  |  |  |  |  |  |  |  |  |  |  |  |  |  |
|  | *S2* | *S.* *major* Kützing ex Gomont |  |  |  |  |  |  | **0.20** |  |  |  |  |  |  |  | **0.02** |  |  | **0.06** |  |  |
|  | *S2* | *S. meneghiniana* Zanardini ex Gomont | **0.04** |  |  |  |  |  |  |  |  |  |  |  |  |  |  |  |  |  |  |  |
|  | *S2* | *S. subsalsa* Oersted ex Gomont |  |  |  |  | **0.37** |  |  |  |  |  |  |  |  |  |  |  |  |  |  |  |
|  | *K* | *Synechocystis sallensis* Skuja |  |  |  |  |  |  | **0.01** |  |  |  |  |  |  |  |  |  |  | **0.00** |  |  |
|  |  | **Diatoms** |  |  |  |  |  |  |  |  |  |  |  |  |  |  |  |  |  |  |  |  |
| **Ach arm** | *MP* | ***Achnanthes* *armillaris* (O.F.Müller) Guiry** | **74.68** | **12.45** | **37.34** |  |  |  |  |  | **248.92** |  |  |  |  |  |  |  |  | **12.45** |  |  |
|  | *MP* | *A.* sp. |  |  |  |  |  |  |  |  |  |  |  |  |  |  | **0.67** |  |  |  |  |  |
|  | *MP* | *Achnanthidium minutissimum* (Kützing) Czarnecki | **1.16** |  |  |  |  |  |  |  |  | **2.32** |  |  |  |  |  |  |  |  |  |  |
| **Am nor** | *MP* | ***Amphora* *normanii* Rabenhorst** |  |  |  |  |  |  | **42.90** | **21.45** | **85.80** |  |  |  | **150.15** |  | **42.90** |  |  |  |  |  |
|  | *MP* | *Amphora* sp. | **184.64** | **46.16** |  |  |  |  |  |  |  |  |  |  |  | **138.48** |  |  |  |  |  |  |
| **Aul gra** | *P* | ***Aulacoseira granulata* (Ehrenberg) Simonsen** |  |  |  |  | **17.86** |  | **17.86** | **11.90** | **23.81** |  |  |  |  |  |  |  |  |  |  |  |
|  | *D* | *Bacillaria paxillifera (O.F.Müller) T.Marsson* |  |  |  |  |  |  |  | **26.65** |  |  |  |  |  |  |  |  |  |  |  |  |
|  | *MP* | *Caloneis* *permagna* (Bailey) Cleve |  |  |  |  |  |  |  |  |  |  |  |  |  | **43.05** |  |  |  |  |  |  |
|  | *MP* | *Campylodiscus neofastuosus* Ruck & Nakov | **8.24** |  |  |  |  |  |  |  |  |  |  |  |  |  |  |  |  |  |  |  |
| **Cer pel** | *P* | ***Cerataulina pelagica*(Cleve) Hendey** |  |  |  |  |  |  |  |  |  |  |  |  | **48.11** |  |  |  | **38.48** | **38.48** |  | **96.21** |
|  | *C* | *Chaetoceros tortissimus* Gran |  |  |  |  |  |  |  |  |  |  |  |  |  |  |  |  | **45.62** |  |  |  |
|  | *C* | *Chaetoceros wighamii* Brightwell |  |  |  |  |  |  |  |  |  |  |  |  |  |  |  |  | **15.92** |  |  |  |
|  | *C* | *Cyclotella meneghiniana Kützing* | **19.01** |  |  |  |  |  |  | **38.01** |  |  |  |  |  |  |  |  |  |  |  |  |
|  | *D* | *Cymbella* sp. |  |  |  |  |  |  |  |  |  |  | **105.23** |  |  |  |  |  | **21.05** |  |  |  |
| **Den ten** | *MP* | ***Denticula tenuis* Kützing** | **15.11** | **40.30** | **25.19** | **65.48** | **20.15** |  |  | **20.15** | **60.45** | **10.07** | **10.07** | **171.26** | **65.48** |  | **25.19** | **65.48** | **25.19** | **952.01** | **5.04** | **55.41** |
| **Dip ob** | *MP* | ***Diploneis* *oblongella* (Nägeli ex Kützing) A.Cleve** |  |  | **101.66** |  |  | **50.83** |  |  |  |  |  | **50.83** |  |  |  | **101.66** |  |  |  |  |
|  | *MP* | *Fragilaria construens* (Ehrenberg) Grunow (ab) | **1.58** |  |  |  | **1.58** |  | **3.17** |  |  |  |  |  |  |  |  |  |  |  |  |  |
|  | *MP* | *Fragillariopsis* sp. |  |  |  |  |  |  |  |  |  | **7.69** |  |  |  |  |  |  |  |  |  |  |
|  | *MP* | *Gomphonema subtile* Ehrenberg |  |  |  |  |  |  |  | **23.36** |  |  |  |  |  |  |  |  |  |  |  |  |
|  | *MP* | *Mastogloia erythraea* Grunow |  |  |  |  |  |  |  |  |  | **5.13** |  |  |  |  |  |  |  |  |  |  |
| **Mas br** | *MP* | ***M. braunii* Grunow** | **197.81** | **65.94** |  |  | **263.74** |  | **395.62** |  | **263.74** |  | **2769.32** |  |  | **131.87** | **65.94** |  |  | **263.74** | **65.94** |  |
| **Mel mon** | *P* | ***Melosira moniliformis* C.Agardh** | **5569.69** | **322.88** |  |  |  |  |  | **726.48** | **23893.18** |  | **161.44** |  |  |  |  |  |  |  |  |  |
| **Nav er** | *MP* | ***Navicula erifuga* Lange-Bertalot** | **48.26** | **24.13** |  |  |  |  |  |  | **96.52** |  | **337.83** | **48.26** |  |  |  |  |  |  |  |  |
|  | *MP* | *Navicula perrhombus* Hustedt ex Simonsen |  |  |  |  |  |  |  | **214.88** |  |  |  |  |  |  |  |  |  |  |  |  |
|  | *MP* | *N.* sp. |  |  |  |  |  |  |  | **44.50** |  |  |  |  |  |  |  |  |  |  |  |  |
|  | *MP* | *N.* sp.1 |  |  |  |  |  |  |  |  | **116.68** |  | **29.17** | **14.59** |  |  |  |  |  |  |  |  |
|  | *D* | *Nitzschia acicularioides* Hustedt |  |  |  |  |  |  |  |  |  |  |  |  |  |  |  |  |  |  |  |  |
|  | *D* | *N. closterium* (Ehrenberg) W.Smith |  | **0.26** |  |  |  |  |  |  |  |  |  |  |  |  |  |  |  |  |  |  |
|  | *D* | *N. filiformis* (W.Smith) Van Heurck |  |  |  |  |  |  |  |  |  |  |  |  |  |  |  |  |  |  |  |  |
|  | *D* | *N. fonticola* (Grunow) Grunow |  |  |  |  |  |  |  |  |  |  |  | **3.04** | **4.56** | **24.30** |  |  |  |  |  |  |
|  | *D* | *N. longissima* (Brébisson) Ralfs |  |  |  |  |  |  |  |  | **30.00** |  |  |  |  |  |  | **67.50** |  |  |  |  |
| **Nit pal** | *D* | ***N. palea* (Kützing) W.Smith** | **29.84** | **3.73** |  |  | **52.22** |  |  | **3.73** |  |  |  | **26.11** |  |  |  |  |  |  |  |  |
|  | *D* | *N. paleacea* (Grunow) Grunow |  |  | **2.74** |  |  |  |  |  |  |  |  |  |  |  |  |  |  |  |  |  |
| **Nit sig** | *D* | ***N. sigma* (Kützing) W.Smith** | **251.47** | **100.59** | **100.59** | **50.29** | **201.18** |  | **251.47** |  |  | **301.77** | **5431.83** |  | **201.18** |  | **50.29** |  |  |  | **502.95** | **50.29** |
|  | *MP* | *N. sigmoidea* (Nitzsch) W.Smith | **284.95** |  |  |  |  |  | **47.49** |  | **94.98** |  |  |  |  |  |  |  |  |  |  |  |
| **Nit sp** | *D* | ***N.* sp.** | **10.53** |  |  | **6.32** | **21.06** |  | **82.34** |  | **67.39** | **33.69** | **33.69** | **2.11** | **84.23** | **71.60** |  | **8.42** |  | **12.63** |  | **50.54** |
|  | *D* | *N.* sp.1 |  |  |  |  |  |  |  |  | **74.67** |  | **179.20** |  |  |  |  |  |  |  |  |  |
| **Pan oce** | *B* | ***Pantocsekiella ocellata* (Pantocsek) K.T.Kiss & Ács** | **2.06** | **4.11** |  |  |  | **8.22** |  | **4.11** |  |  |  |  |  |  |  |  |  |  |  | **2.06** |
| **Ple el** | *MP* | ***Pleurosigma elongatum W.Smith*** | **9908.31** | **471.82** |  |  |  |  |  |  | **4718.24** |  |  |  |  | **943.65** |  |  |  |  |  |  |
|  | *MP* | *Rhobalodia* sp. |  |  |  |  |  | **48.72** |  |  | **487.23** |  |  |  |  |  |  |  |  |  |  |  |
|  | *MP* | *Sellaphora pupula* (Kützing) Mereschkovsky |  |  |  |  |  |  |  |  |  |  |  |  | **8.18** |  |  |  |  |  |  |  |
| **Shi oes** | *B* | ***Shionodiscus* *oestrupii* (Ostenfeld) A.J.Alverson, S.-H.Kang & E.C.Theriot** | **6.54** | **0.38** |  |  |  | **0.77** |  |  | **2.31** |  |  |  |  | **6.93** |  |  |  |  | **0.38** |  |
| **Tet dec** | *MP* | ***Tetramphora decussata* (Grunow) Stepanek & Kociolek** | **949.26** | **9.13** |  | **91.27** | **1223.08** | **155.17** | **5503.86** | **1907.64** | **492.88** | **8561.56** | **219.06** | **36.51** | **73.02** | **18.25** | **63.89** | **27.38** | **155.17** | **45.64** | **27.38** |  |
|  | *MP* | *Ulnaria ulna* (Nitzsch) Compère |  |  |  |  |  |  |  |  | **133.83** | **133.83** |  |  |  |  |  |  |  |  |  |  |
|  |  | **Chlorophyceae** |  |  |  |  |  |  |  |  |  |  |  |  |  |  |  |  |  |  |  |  |
|  | *N* | *Cosmarium montrealense Croasdale* |  |  |  |  |  | **0.38** |  |  |  |  |  |  |  | **1.54** |  |  |  |  |  |  |
| **Mon sim** | *J* | ***Monactinus* *simplex* (Meyen) Corda** | **15.39** |  |  |  |  | **3.08** |  | **10.78** | **12.31** | **12.31** |  |  |  |  |  |  |  |  |  |  |
|  | *X1* | *Monoraphidium contortum* (Thuret) Komárková-Legnerová |  |  |  |  |  |  |  |  |  |  |  |  |  | **12.70** |  |  |  |  |  |  |
|  | *P* | *Staurastrum curviceps* A.M.Scott & Grönblad | **0.38** |  |  |  |  |  |  |  |  |  |  |  |  |  |  |  |  |  |  |  |
|  |  | **Prymnesiophyceae** |  |  |  |  |  |  |  |  |  |  |  |  |  |  |  |  |  |  |  |  |
|  | *X3* | *Phaeocystis globosa* Scherffel |  |  |  |  |  |  |  |  |  |  |  |  |  |  |  | **2.69** |  |  |  |  |
|  |  | **Dino** |  |  |  |  |  |  |  |  |  |  |  |  |  |  |  |  |  |  |  |  |
|  | *Y* | *Archaeperidinium minutum (Kofoid) Jørgensen* |  |  |  |  |  |  |  | **0.77** |  |  |  |  |  |  |  |  |  |  |  |  |
|  | *Lo* | *Protodinium* *simplex* Lohmann |  |  |  |  |  |  |  | **0.77** |  |  |  | **0.38** |  |  |  |  |  |  |  | **0.38** |
|  | *Lo* | *Protoperidinium ventricum (T.H.Abé) Balech* |  |  |  |  |  |  |  |  | **2.31** |  |  |  |  |  |  |  |  |  |  |  |

Table S1 Mean Bio-volume of different Phytoplankton species (µm^3^ x 10^6^ / L) at different drains and wells, with the corresponding functional groups and the abbreviations of the most frequent species used in the RDA analysis, (D_1-3_) and wells (W_1-17_)

| Indicator species | Cluster 1 | |  |  | Cluster 2 |  | |  | | Cluster 3 |  | |  | | Cluster 4 |  | |
| --- | --- | --- | --- | --- | --- | --- | --- | --- | --- | --- | --- | --- | --- | --- | --- | --- | --- |
|  | F % | A % | IndVal |  | F % | A % | IndVal | |  | F % | A % | IndVal | |  | F % | A % | IndVal |
| *Gomphosphaeria sp.* | 75 | 0.31 | 16.35 |  | 50 | 0.31 | 10.68 | |  | 50 | 0.40 | 13.86 | |  | 33.33 | 0.42 | 9.706 |
| *Achnanthes armillaris* | 75 | 0.59 | 9.91 |  | 0 | 0.00 | 0 | |  | 0 | 0.00 | 0 | |  | 33.33 | 3.86 | 28.93 |
| *Amphora sp.* | 75 | 3.76 | 75 |  | 0 | 0.00 | 0 | |  | 0 | 0.00 | 0 | |  | 0 | 0 | 0 |
| *Cerataulina pelagica* | 0 | 0.00 | 0 |  | 16.67 | 1.55 | 2.73 | |  | 0 | 0.00 | 0 | |  | 50 | 7.91 | 41.82 |
| *Cymbella sp.* | 0 | 0.00 | 0 |  | 16.67 | 0.85 | 13.62 | |  | 0 | 0.00 | 0 | |  | 16.67 | 0.19 | 3.05 |
| *Denticula tenuis* | 75 | 0.98 | 1.4 |  | 66.67 | 1.34 | 1.69 | |  | 100 | 30.81 | 58.55 | |  | 100 | 19.49 | 37.04 |
| *Diploneis oblongella* | 0 | 0.00 | 0 |  | 33.33 | 4.55 | 10.41 | |  | 25 | 8.05 | 14.9 | |  | 16.67 | 1.24 | 1.53 |
| *M. braunii* | 100 | 4.32 | 18.04 |  | 33.33 | 3.49 | 4.86 | |  | 25 | 6.14 | 6.41 | |  | 50 | 9.99 | 20.88 |
| *Melosira moniliformis* | 75 | 34.91 | 66.2 |  | 16.67 | 4.28 | 1.83 | |  | 0 | 0.00 | 0 | |  | 16.67 | 0.29 | 0.12 |
| *Navicula erifuga* | 75 | 0.69 | 9.83 |  | 0 | 0.00 | 0 | |  | 25 | 3.98 | 18.85 | |  | 16.67 | 0.61 | 1.92 |
| *N. fonticola* | 25 | 0.43 | 13.57 |  | 0 | 0.00 | 0 | |  | 25 | 0.25 | 7.917 | |  | 16.67 | 0.11 | 2.34 |
| *N. sigma* | 50 | 2.63 | 2.35 |  | 50 | 3.08 | 2.749 | |  | 50 | 8.67 | 7.734 | |  | 83.33 | 41.64 | 61.95 |
| *N. palea* | 50 | 0.13 | 2.273 |  | 33.33 | 0.51 | 6.06 | |  | 25 | 2.16 | 19.32 | |  | 0 | 0 | 0 |
| *N. sp.* | 75 | 1.34 | 10.86 |  | 50 | 0.47 | 2.56 | |  | 75 | 1.85 | 15.04 | |  | 66.67 | 5.56 | 40.22 |
| *Pleurosigma elongatum* | 100 | 45.24 | 100 |  | 0 | 0.00 | 0 | |  | 0 | 0.00 | 0 | |  | 0 | 0 | 0 |
| *Rhobalodia sp.* | 25 | 0.39 | 2.56 |  | 16.67 | 3.43 | 14.96 | |  | 0 | 0.00 | 0 | |  | 0 | 0 | 0 |
| *Tetramphora decussata* | 100 | 2.27 | 2.38 |  | 100 | 69.83 | 73.05 | |  | 100 | 20.01 | 20.93 | |  | 66.67 | 3.48 | 2.43 |

Table S2. Mean percentage of abundance (A%), frequency percentage (F%) and indicator values (IndVal) of different indicator species at different clusters of phytoplanktonic indicator species at *P* <0.01

| Abbreviation of species used in the RDA analysis | Zooplankton Species | **D1** | **D2** | **D3** | **W1** | **W2** | **W3** | **W4** | **W5** | **W6** | **W7** | **W8** | **W9** | **W10** | **W11** | **W12** | **W13** | **W14** | **W15** | **W16** | **W17** |
| --- | --- | --- | --- | --- | --- | --- | --- | --- | --- | --- | --- | --- | --- | --- | --- | --- | --- | --- | --- | --- | --- |
|  | **Rotifer** |  |  |  |  |  |  |  |  |  |  |  |  |  |  |  |  |  |  |  |  |
|  | *Asplanchna priodonta* Gosse | **0** | **0** | **0** | **0** | **0** | **0** | **0** | **0** | **667** | **0** | **0** | **0** | **0** | **0** | **0** | **0** | **0** | **0** | **0** | **0** |
|  | *Ascomorpha agilis* Zacharias | **0** | **667** | **0** | **0** | **0** | **0** | **0** | **0** | **0** | **0** | **0** | **0** | **0** | **0** | **0** | **0** | **0** | **0** | **0** | **0** |
|  | *Brachionus angularis* Gosse | **0** | **0** | **0** | **0** | **0** | **0** | **0** | **0** | **667** | **0** | **0** | **0** | **0** | **0** | **0** | **0** | **0** | **0** | **0** |  |
|  | *B. calyciflorus* Pallas | **0** | **0** | **0** | **0** | **0** | **667** | **0** | **0** | **0** | **0** | **0** | **0** | **0** | **0** | **0** | **0** | **0** | **0** | **0** | **0** |
|  | *B. quadridentatus* Hermann | **0** | **0** | **0** | **0** | **0** | **0** | **0** | **0** | **0** | **0** | **0** | **0** | **0** | **0** | **667** | **0** | **0** | **0** | **0** |  |
|  | *B. urceolaris* Müller | **0** | **0** | **0** | **0** | **0** | **0** | **667** | **1334** | **0** | **0** | **0** | **0** | **0** | **0** | **0** | **0** | **0** | **0** | **0** | **0** |
| **Ceph gib** | ***Cephalodella gibba* Ehrenberg** | **0** | **0** | **0** | **667** | **667** | **0** | **0** | **0** | **0** | **667** | **0** | **0** | **0** | **0** | **0** | **0** | **0** | **0** | **0** | **0** |
|  | *Collotheca ornata* Ehrenberg | **0** | **0** | **0** | **0** | **0** | **0** | **667** | **667** | **0** | **0** | **0** | **0** | **0** | **0** | **0** | **0** | **0** | **0** | **0** | **0** |
| **Col ad** | ***Colurella adriatica* Ehrenberg** | **3000** | **1334** | **667** | **0** | **3335** | **1334** | **5336** | **3335** | **5336** | **2668** | **667** | **2001** | **2001** | **0** | **667** | **667** | **2001** | **2668** | **0** | **667** |
| **Con uni** | ***Conochilus unicornis* Rousselet** | **0** | **0** | **0** | **0** | **0** | **0** | **0** | **1334** | **0** | **0** | **0** | **0** | **0** | **0** | **667** | **0** | **0** | **0** | **0** | **0** |
|  | *Euchlanis dilatata* Ehrenberg | **0** | **0** | **0** | **0** | **0** | **0** | **0** | **0** | **0** | **0** | **0** | **0** | **0** | **0** | **667** | **0** | **0** | **0** | **0** | **0** |
|  | *Filinia longiseta* Ehrenberg | **0** | **0** | **0** | **0** | **667** | **0** | **0** | **0** | **0** | **0** | **0** | **0** | **0** | **0** | **0** | **0** | **0** | **0** | **0** | **0** |
|  | *F. terminalis* Plate | **0** | **0** | **0** | **0** | **0** | **0** | **0** | **0** | **0** | **0** | **0** | **0** | **0** | **0** | **0** | **0** | **0** | **0** | **667** | **0** |
| **Hex sp** | ***Hexarthra* sp.** | **0** | **0** | **667** | **0** | **0** | **0** | **0** | **667** | **2668** | **2001** | **0** | **0** | **0** | **1334** | **667** | **0** | **0** | **0** | **0** | **0** |
| **Ker coch** | ***Keratella cochlearis* Gosse** | **1000** | **667** | **0** | **667** | **0** | **667** | **667** | **2668** | **1334** | **2668** | **667** | **0** | **0** | **0** | **667** | **0** | **667** | **0** | **667** |  |
| **Lec bul** | ***Lecane bulla* Gosse** | **6000** | **6670** | **667** | **2001** | **2668** | **0** | **3335** | **667** | **3335** | **6003** | **2001** | **10005** | **4002** | **4669** | **1334** | **1334** | **0** | **2001** | **1334** | **0** |
| **Lec clos** | ***L. closterocerca* Schmarda** | **1000** | **667** | **0** | **1334** | **0** | **0** | **667** | **667** | **0** | **2001** | **0** | **667** | **0** | **667** | **0** | **0** | **0** | **0** | **0** |  |
| **Lec sp** | ***L.* sp.** | **0** | **667** | **0** | **2001** | **667** | **1334** | **667** | **0** | **0** | **2668** | **3335** | **3335** | **2668** | **3335** | **0** | **0** | **0** | **1334** | **667** |  |
| **Lep pat** | ***Lepadella patella* Müller** | **0** | **0** | **0** | **0** | **0** | **0** | **0** | **0** | **667** | **0** | **0** | **0** | **0** | **0** | **0** | **0** | **0** | **0** | **0** |  |
|  | *Macrochaetus subqudratus* Perty | **0** | **0** | **0** | **0** | **667** | **667** | **0** | **0** | **0** | **0** | **667** | **2001** | **667** | **667** | **0** | **0** | **0** | **667** | **0** | **0** |
|  | *Notholca acuminata*Ehrenberg | **0** | **0** | **0** | **0** | **0** | **0** | **0** | **667** | **0** | **0** | **0** | **0** | **667** | **0** | **0** | **0** | **0** | **0** | **0** |  |
| **Phil ros** | ***Philodina roseola* Ehrenberg** | **0** | **0** | **667** | **0** | **0** | **0** | **0** | **0** | **0** | **0** | **0** | **0** | **0** | **0** | **0** | **0** | **0** | **0** | **0** |  |
| **Trich el** | ***Trichocerca elongata* Gosse** | **0** | **0** | **0** | **0** | **0** | **0** | **0** | **0** | **667** | **1334** | **0** | **0** | **0** | **0** | **6670** | **2001** | **0** | **0** | **0** | **0** |
|  | *T. pusilla* Jennings | **0** | **0** | **0** | **1334** | **0** | **0** | **0** | **0** | **0** | **0** | **0** | **0** | **0** | **0** | **0** | **0** | **0** | **0** | **0** | **0** |
|  | **Copepoda** |  |  |  |  |  |  |  |  |  |  |  |  |  |  |  |  |  |  |  |  |
| **Naup L** | **Nauplius larvae** | **0** | **667** | **0** | **0** | **667** | **667** | **667** | **2001** | **667** | **10672** | **667** | **0** | **667** | **1334** | **0** | **0** | **0** | **0** | **0** | **667** |
| **Cop st** | **Copepodite stage** | **0** | **0** | **0** | **0** | **0** | **0** | **667** | **0** | **1334** | **1334** | **0** | **0** | **0** | **0** | **0** | **0** | **0** | **0** | **0** | **0** |
|  | *Mesocyclops* sp. | **0** | **0** | **0** | **0** | **0** | **0** | **667** | **0** | **0** | **667** | **0** | **0** | **0** | **0** | **0** | **0** | **0** | **0** | **0** |  |
|  | **Protozoa** |  |  |  |  |  |  |  |  |  |  |  |  |  |  |  |  |  |  |  |  |
|  | *Arcella arenaria* Greeff | **0** | **0** | **0** | **0** | **0** | **0** | **0** | **0** | **2668** | **1334** | **0** | **0** | **0** | **0** | **0** | **0** | **0** | **0** | **0** | **0** |
|  | *Acineta tuberosa* Ehrenberg | **0** | **2001** | **0** | **0** | **0** | **0** | **0** | **0** | **0** | **0** | **0** | **0** | **0** | **0** | **0** | **0** | **0** | **0** | **0** | **0** |
|  | **Meroplankton** |  |  |  |  |  |  |  |  |  |  |  |  |  |  |  |  |  |  |  |  |
| **Nem lar** | **Nematoda larvae** | **2000** | **667** | **0** | **0** | **1334** | **0** | **0** | **667** | **4669** | **667** | **667** | **667** | **667** | **667** | **0** | **0** | **667** | **1334** | **667** | **0** |
|  | Chironomus larvae | **0** | **0** | **0** | **0** | **0** | **0** | **0** | **0** | **4002** | **0** | **0** | **0** | **0** | **667** | **0** | **0** | **0** | **0** | **0** | **0** |
| **Ena sp** | **Nymph of *Enallagma* sp.** | **0** | **0** | **0** | **0** | **2001** | **0** | **0** | **0** | **0** | **0** | **0** | **0** | **1334** | **0** | **0** | **0** | **0** | **0** | **0** | **0** |

Table S3. Mean density of different Zooplankton species at different drains and wells (Org. m^-3^) and the abbreviations of the most frequent species used in the RDA analysis, (D_1-3_) and wells (W_1-17_)

| Indicator species | Cluster 1 | |  |  | Cluster 2 | |  |  | Cluster 3 | |  |  | Cluster 4 | |  |
| --- | --- | --- | --- | --- | --- | --- | --- | --- | --- | --- | --- | --- | --- | --- | --- |
|  | F % | A % | IndVal |  | F % | A % | IndVal |  | F % | A % | IndVal |  | F % | A % | IndVal |
| *Brachionus urceolaris* | 14.29 | 1.30 | 8.24 |  | 20 | 0.95 | 8.46 |  | 0 | 0.00 | 0.00 |  | 0 | 0.00 | 0.00 |
| *Cephalodella gibba* | 14.29 | 0.27 | 1.45 |  | 20 | 1.05 | 7.75 |  | 16.67 | 1.39 | 8.52 |  | 0 | 0.00 | 0.00 |
| *Collotheca ornata* | 14.29 | 0.65 | 5.79 |  | 20 | 0.95 | 11.89 |  | 0 | 0.00 | 0.00 |  | 0 | 0.00 | 0.00 |
| *Colurella adriatica* | 100 | 26.30 | 37.90 |  | 100 | 27.32 | 39.38 |  | 50 | 4.66 | 3.36 |  | 100 | 11.11 | 16.01 |
| *Conochilus unicorni* | 14.29 | 1.30 | 4.55 |  | 0 | 0.00 | 0.00 |  | 0 | 0.00 | 0.00 |  | 50 | 2.78 | 34.07 |
| *Hexarthra sp.* | 57.14 | 6.38 | 33.68 |  | 0 | 0.00 | 0.00 |  | 16.67 | 1.67 | 2.57 |  | 50 | 2.78 | 12.83 |
| *Keratella cochlearis* | 85.71 | 12.58 | 44.75 |  | 40 | 2.49 | 4.14 |  | 66.67 | 6.24 | 17.28 |  | 50 | 2.78 | 5.77 |
| *Lecane bulla* | 57.14 | 1.47 | 4.96 |  | 100 | 2.49 | 30.63 |  | 100 | 5.00 | 37.63 |  | 100 | 0.00 | 23.06 |
| *L. closterocerca* | 28.57 | 8.24 | 4.70 |  | 40 | 9.55 | 11.11 |  | 66.67 | 21.29 | 37.18 |  | 0 | 0.00 | 0.00 |
| *Lecane bulla* | 42.86 | 1.79 | 9.04 |  | 80 | 3.77 | 19.55 |  | 100 | 3.90 | 54.48 |  | 0 | 0.00 | 0.00 |
| *Macrochaetus subqudratu* | 14.29 | 0.65 | 2.70 |  | 60 | 1.05 | 23.93 |  | 50 | 0.00 | 20.62 |  | 0 | 0.00 | 0.00 |
| *Philodina roseola* | 14.29 | 8.36 | 5.45 |  | 20 | 29.52 | 12.37 |  | 0 | 36.27 | 0.00 |  | 0 | 22.22 | 0.00 |
| *Trichocerca elongata* | 28.57 | 0.88 | 0.47 |  | 0 | 0.00 | 0.00 |  | 0 | 0.00 | 0.00 |  | 100 | 52.78 | 98.36 |
| *Nauplius larvae* | 71.43 | 12.03 | 45.64 |  | 60 | 3.06 | 9.74 |  | 50 | 3.74 | 9.94 |  | 0 | 0.00 | 0.00 |
| *Copepodite stage* | 28.57 | 1.22 | 16.02 |  | 20 | 0.95 | 8.79 |  | 0 | 0.00 | 0.00 |  | 0 | 0.00 | 0.00 |
| *Mesocyclops sp.* | 14.29 | 0.27 | 3.20 |  | 20 | 0.95 | 15.52 |  | 0 | 0.00 | 0.00 |  | 0 | 0.00 | 0.00 |
| *Arcella arenaria* | 28.57 | 1.88 | 28.57 |  | 0 | 0.00 | 0.00 |  | 0 | 0.00 | 0.00 |  | 0 | 0.00 | 0.00 |
| *Nematoda larvae* | 57.14 | 6.11 | 15.91 |  | 80 | 9.57 | 34.85 |  | 83.33 | 6.28 | 23.84 |  | 0 | 0.00 | 0.00 |
| *Chironomus larvae* | 14.29 | 2.00 | 10.08 |  | 0 | 0.00 | 0.00 |  | 16.67 | 0.71 | 4.90 |  | 0 | 0.00 | 0.00 |
| *Nymph of Enallagma sp.* | 0 | 0.00 | 0.00 |  | 40 | 5.26 | 40.00 |  | 0 | 0.00 | 0.00 |  | 0 | 0.00 | 0.00 |

Table S4. Mean abundance percentage (A%) and frequency percentage (F%) and indicator values (IndVal) of different indicator species at different clusters of zooplanktonic indicator species at *P* <0.01

| Variables | TSI_N_ | TSI_BIO_ | TSI_BAC_ | TSI_B/N_ | TSI_IHT_ | TSI_ROT_ | Phytoplankton index (Q) |
| --- | --- | --- | --- | --- | --- | --- | --- |
| TSI_N_ | **1** | **0.756** | **-0.444** | -0.200 | -0.302 | **0.736** | **0.610** |
| TSI_BIO_ | **0.756** | **1** | -0.266 | **-0.789** | -0.050 | **0.844** | **0.640** |
| TSI_BAC_ | **-0.444** | -0.266 | **1** | -0.035 | **0.799** | 0.155 | -0.085 |
| TSI_B/N_ | -0.200 | **-0.789** | -0.035 | **1** | -0.237 | **-0.589** | -0.202 |
| TSI_IHT_ | -0.302 | -0.050 | **0.799** | -0.237 | **1** | 0.337 | 0.168 |
| TSI_ROT_ | **0.736** | **0.844** | 0.155 | **-0.589** | 0.337 | **1** | **0.697** |
| Phytoplankton index (Q) | **0.610** | **0.640** | -0.085 | -0.402 | 0.168 | **0.697** | **1** |
| Temperature | -0.390 | -0.398 | **0.518** | 0.189 | 0.196 | -0.211 | -0.386 |
| Turbidity | 0.345 | **0.515** | 0.032 | **-0.463** | 0.072 | **0.489** | 0.280 |
| TIN | **0.572** | **0.494** | 0.296 | 0.219 | 0.223 | **0.421** | **-0.435** |
| pH | 0.163 | 0.250 | -0.368 | -0.220 | -0.243 | 0.032 | -0.116 |
| HCO_3_ | -0.043 | -0.082 | -0.064 | 0.133 | -0.098 | -0.107 | 0.007 |
| Salinity | -0.193 | -0.289 | -0.126 | 0.297 | -0.119 | -0.325 | 0.102 |
| BOD | 0.089 | 0.233 | -0.324 | -0.284 | 0.041 | 0.072 | **0.482** |
| COD | **0.631** | 0.435 | -0.161 | -0.076 | -0.170 | **0.486** | 0.205 |
| DO | 0.052 | 0.382 | -0.326 | **-0.515** | -0.212 | 0.056 | 0.059 |
| TP | -0.220 | -0.272 | -0.146 | 0.261 | -0.164 | -0.350 | 0.010 |
| PO_4_ | -0.220 | -0.272 | -0.146 | 0.261 | -0.164 | -0.350 | 0.010 |
| SO_4_ | -0.229 | -0.210 | -0.107 | 0.159 | -0.116 | -0.299 | 0.091 |
| Cl | -0.150 | -0.280 | -0.141 | 0.319 | -0.133 | -0.305 | 0.128 |
| Ca | -0.195 | -0.244 | -0.034 | 0.218 | -0.015 | -0.247 | 0.204 |
| Mg | -0.272 | -0.360 | -0.048 | 0.332 | -0.108 | -0.379 | 0.074 |
| Na | -0.139 | -0.265 | -0.178 | 0.313 | -0.144 | -0.306 | 0.088 |
| K | -0.037 | -0.157 | -0.241 | 0.245 | -0.212 | -0.232 | 0.205 |
| NO_3_ | **0.633** | **0.534** | 0.333 | 0.225 | 0.250 | **0.457** | **-0.409** |
| NH_4_ | 0.346 | 0.225 | -0.211 | -0.034 | -0.161 | 0.199 | 0.285 |

Table S5 Correlation of Q and TSI_ROT_ indices with different environmental variables, Values in bold are significance at *P* < 0.05. Explanation of different abbreviations are represented in table 3 in the main manuscript

| **Site No.** | **Native Name** | **Latitude** | **Longitude** |
| --- | --- | --- | --- |
|  | **Drains** | | |
| D1 | El Qarabeen El Omumy Drain | 29°12'33.8" | 25°30'16.2" |
| D2 | Gheet El Khalel Drain | 29°11'37.0" | 25°34'06.7" |
| D3 | El-Omumy Drain | 29°10'05.7" | 25°45'44.9" |
|  | **Wells** | | |
| W1 | Telwa | 29°13'58.7" | 25°28'41.9" |
| W2 | Kharafala | 29°14'19.9" | 25°30'33.8" |
| W3 | Mohamed Belal | 29°10'58.1" | 25°29'30.6" |
| W4 | El-Shaheem | 29°10'51.6" | 25°29'43.9" |
| W5 | Tegzerti I | 29°11'14.6" | 25°29'43.3" |
| W6 | Tegzerti II | 29°11'14.9" | 25°29'41.6" |
| W7 | Fentas | 29°12'01.1" | 25°30'46.4" |
| W8 | Cleopatra II | 29°11'42.5" | 25°32'47.3" |
| W9 | Telham | 29°11'44.9" | 25°32'58.6" |
| W10 | Cleopatra I | 29°11'54.1" | 25°33'00.2" |
| W11 | El-Molouk | 29°11'04.9" | 25°33'15.8" |
| W12 | Korisht I | 29°12'38.9" | 25°42'21.2" |
| W13 | Korisht II | 29°12'24.5" | 25°42'42.2" |
| W14 | El-Naqb I | 29°10'35.7" | 25°45'48.4" |
| W15 | El-Naqb II | 29°10'43.8" | 25°46'09.8" |
| W16 | Hayat | 29°08'39.0" | 25°43'59.1" |
| W17 | Zeitoun | 29°09'53.5" | 25°47'16.8" |

Table S6 Collected samples from the main drains (D1-D3) and wells (W1-W17) in Siwa Oasis with their native names and Lat and Long.
